# Macroporosity Control by Phase Separation in Sol-Gel Derived Monoliths and Microspheres

**DOI:** 10.3390/ma14154247

**Published:** 2021-07-29

**Authors:** Ana C. Marques, Mário Vale

**Affiliations:** CERENA, DEQ, Instituto Superior Técnico, Universidade de Lisboa, Avenida Rovisco Pais, 1049-001 Lisboa, Portugal; info@cerena.tecnico.ulisboa.pt

**Keywords:** macroporosity, interconnected macropores, microspheres, monoliths, hierarchical porosity, phase separation, sol-gel, spinodal decomposition, silica, non-oxides

## Abstract

Macroporous and hierarchically macro/mesoporous materials (mostly monoliths and microspheres) have attracted much attention for a variety of applications, such as supporting or enabling materials in chromatography, energy storage and conversion, catalysis, biomedical devices, drug delivery systems, and environmental remediation. A well-succeeded method to obtain these tailored porous materials relies on the sol-gel technique, combined with phase separation by spinodal decomposition, and involves as well emulsification as a soft template, in the case of the synthesis of porous microspheres. Significant advancements have been witnessed, in terms of synthesis methodologies optimized either for the use of alkoxides or metal–salts and material design, including the grafting or immobilization of a specific species (or nanoparticles) to enable the most recent trends in technological applications, such as photocatalysis. In this context, the evolution, in terms of material composition and synthesis strategies, is discussed in a concerted fashion in this review, with the goal of inspiring new improvements and breakthroughs in the framework of porous materials.

## 1. Introduction and Motivation

Tailored porous materials with controlled pore size, shape, and morphology (including pore interconnectivity) are a relevant research topic in the materials and chemistry domains, due to their high potential as porous support materials, for sustainable and innovative applications in the fields of catalysis, chromatography, controlled release, scaffolds for biomedical applications, sensing, energy storage and conversion, sorption, and separation. Research on such complex, often hierarchically organized, porous materials has tremendously progressed in the last decades [1], involving varied strategies for creating porosity at different scales within monolithic structures, powders, or, more recently, controlled diameter microspheres [2], which may be preferred since they can be easily packed into existing reactors, columns, or other containers.

The synthesis and development of porous microspheres for the photocatalytic purification of water (driven by the United Nations Sustainable Development Goals) is an example of the immense applications of porous support materials [3]. In this case, porosity directly influences the photocatalytic activity, since macropores (>50 nm) facilitate the mass transport of liquids and gases to the active sites, giving better performance in flow-through catalytic systems; mesopores (2–50 nm) are responsible for the high surface area of these materials, as well as for the size and shape selectivity.

Solution-based strategies, including the co-assembly of molecular precursors with “soft templating”, such as surfactant, emulsion, or breath figure templating are often employed in the synthesis of hierarchically organized porous materials. “Hard templating” approaches, such as colloidal, polymer, or bio-templating, can also be employed [4]. In these cases, a post template-removal process is necessary, which makes them costly and does not facilitate large-scale production. Hierarchically organized porous materials can also be synthesized by template-free methods, such as spontaneous self-formation, sol-gel controlling, and selective leaching [4]. These are simpler than templating approaches; however, the size and shape of the obtained pores are more difficult to control. One example of materials obtained via the sol-gel/carbonization process is hierarchical titania (TiO_2_)/carbon (C) nanocomposite monoliths with a robust scaffolding architecture, which display a macro/mesoporous network and TiO_2_-C heterostructure for high-performance lithium-ion batteries [5].

The sol-gel/phase separation method consists of a solution-based approach to control porosity generation within a material, which occurs concurrently with the sol-gel processing of inorganic and organic precursors. Once mastered, it is a very straightforward and relevant method, due to its versatility, lack of need for templates, precise structural control, interconnected porosity, and good reproducibility. From a thermodynamic point of view, phase separation, or the demixing of a system, can be achieved either by physical or chemical cooling. The latter can be driven by the condensation, or polymerization, of a species in a sol-gel system, revealing the importance of the timing of gelation versus phase separation.

Despite the comprehensive reviews on hierarchically porous monoliths that have been published [1,4,6,7], to the best of our knowledge, there are no reviews in the literature on hierarchically porous microspheres achieved by the sol-gel/phase separation method. This review discusses the pioneering and development works, as well as the current state of the art and tendencies in the field of porosity control through sol-gel/phase separation occurring not only in bulk solutions, giving rise to porous monoliths, but also within tiny droplets of a microemulsion, which results in porous microspheres. It focuses on the most influential players (and their corresponding works) that have shaped this field of study. The next sections briefly highlight the fundamentals of phase separation and its combination with sol-gel processing to achieve hierarchically porous materials, followed by the early works on sol-gel/phase separation that were at the origin of this field of study and, finally, more recent key examples for silica-based materials, single- and multi-component oxides, and non-siliceous materials, such as phosphates, titanates, zirconates, metal-organic hybrids, and carbon.

## 2. Background on Phase Separation

In generic terms, phase separation is a spontaneous thermodynamic phenomenon, which results in the creation of at least two distinct phases from a homogeneous mixture. In general, phase separation starts from a molecular length scale and proceeds with the growing of the characteristic size (mean dimension of a set of skeleton and pore) of phase-separated domains with time toward a macroscopic length scale. The dynamics of phase separation were studied intensively in the 20th century [8,9], notwithstanding the recent studies that have been devoted to this topic, namely the coarsening behavior of the network-forming phase separation of colloidal suspensions [10,11]. In particular, phase separation by spinodal decomposition occurs in the unstable region of the miscibility window in an equilibrium phase diagram. In this case, the two developed phases, when in comparable volume fractions, form a 3D interconnected two-phase morphology with a narrow domain size distribution [12], which is the basis for interconnected macroporosity generation.

The thermodynamic process of mixed solutions containing polymers, adapted to the case of macroporous materials generated by phase separation, is described by the Flory-Huggins theory [13,14,15,16], which centers on the expression for the free energy of mixing derived from a lattice model. The theory is constituted by combinatorial entropy terms associated with polymer chain configurations on the lattice, as well as an enthalpic contribution owing to interactions between the different species.

The Gibbs free energy change of mixing, $\Delta G_{m}$, is given by the following equation (Equation (1)):

(1) $$\Delta G_{m}=\;\Delta H_{m}-T\Delta S_{m}\propto RT\left(\frac{\phi_{1}}{P_{1}}ln\phi_{1}+\frac{\phi_{2}}{P_{2}}ln\phi_{2\;}+\chi_{12}\phi_{1}\phi_{2}\right)$$

where *R* is the gas constant (J/mol K), *T* is the temperature (K), $\phi_{i}$ and *P*_i_ are the volume fractions and the degrees of polymerization of the two different components (i = 1 and i = 2) that can be polymerizable (or not (e.g., in the case of solvents)), and $\chi_{12}$ is the Flory interaction parameter, which describes the compatibility between both components.

The first two terms inside the parenthesis in Equation (1) are related to the entropic contribution to $\Delta G_{m}$, and the last term is related to the enthalpic contribution of the system. Therefore, an increase of the polymerization/polycondensation degree, i.e., the formation of non-polar gels (e.g., siloxanes), imposes a decrease in the entropy change ($\Delta S_{m}$) but also an increase in the enthalpy change ($\Delta H_{m}$), due to the arising of polarity differences between the gel and solvent (increased $\chi_{12}$), which results in repulsive interactions. Both will contribute to a gradual increase of $\Delta G_{m}$, possibly from a negative to a positive value, according to Equation (1); the driving force for phase separation is, therefore, generated.

**Figure 1:**
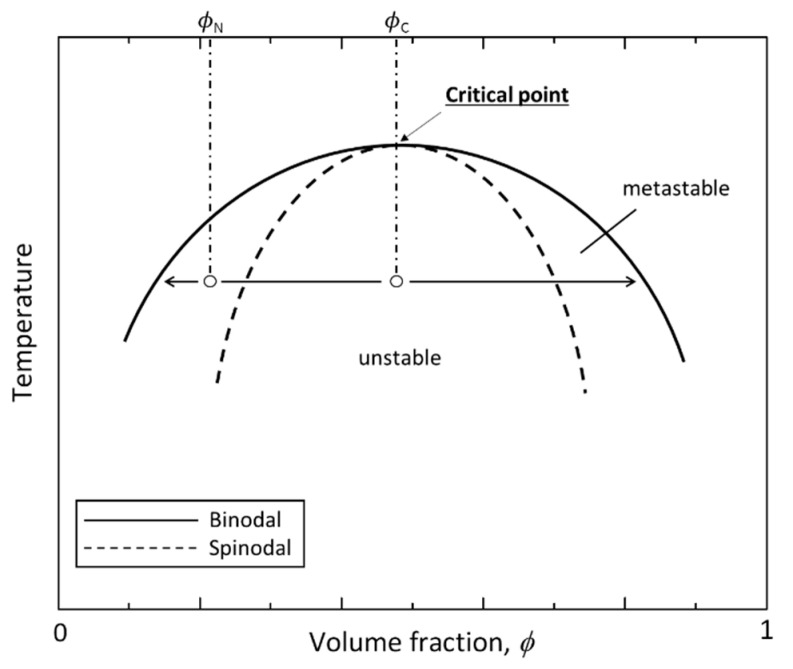
Illustrative phase diagram of a binary system showing UCST behavior. $\phi_{c}$ is the volume fraction of the critical composition, where spinodal decomposition occurs at temperature T_1_. $\phi_{N}$ is the volume fraction of composition, where nucleation-growth occurs at temperature T_1_.

A phase diagram reflecting the phase behavior of any mixture can be made using the Flory-Huggins solution theory (Figure 1), where the binodal and spinodal lines will be reached by solving $\left(\frac{d\Delta G_{m}}{d\phi_{1}}\right)=0$ and $\left(\frac{d^{2}\Delta G_{m}}{d\phi_{1}{}^{2}}\right)=0$, respectively, for the interaction parameter $\chi_{12}$, namely $\chi_{b}$ or $\chi_{s}$. The theoretical definition of $\chi_{12}$ can be used to establish the temperature dependence of polymer miscibility, and the most common experimentally-derived form to demonstrate such dependence is given by Equation (2) [17], which includes temperature-independent terms, i.e., empirical constants A (“entropic part” of $\chi_{12}$) and B, both tabulated for many polymer blends, where $\frac{B}{T}$ is the “enthalpic part” of $\chi_{12}$:

(2) $$\chi_{12}=A+\frac{B}{T}$$

In the phase diagram, the point where binodal and spinodal phases overlap is the critical point or critical value of the Flory interaction parameter. The lower critical solution temperature (LCST) and the upper critical solution temperature (UCST) are the maximum or minimum temperatures, below or above which the components of a mixture are miscible for all compositions, respectively. Depending on the nature of the interaction, the Flory-Huggins theory can predict the phase behavior of mixtures with primarily repulsive (UCST behavior) or attractive (LCST behavior) interactions, namely for a cooling process and for positive values of B. In this case, B > 0, $\chi_{12}$ increases as temperature is lowered (Equation (2)), leading to immiscibility in a UCST system. Figure 1 shows a schematic phase diagram of a general binary system showing UCST behavior. $\phi_{c}$ and $\phi_{N}$ are the volume fractions of the compositions where spinodal decomposition and nucleation-growth happen, respectively, at temperature T_1_.

Critical point, or critical composition ($\phi_{C}$), is given at the point where the binodal and spinodal lines coincide, i.e., in a binary blend showing UCST behavior to the highest point on the spinodal curve, so that it corresponds to $\left(\frac{\partial \chi_{s}}{\partial \phi }\right)=0$. The solution of this equation gives $\phi_{C}$ (Equation (3)), which is found to depend on the degree of the polymerization of both components, related to their number of statistical segments, or lattice sites, $N_{1}$ and $N_{2}$. It should be noted that lattice sites are of the order of monomer sizes, however do not necessarily correspond precisely to the chemical monomer or the Kuhn monomer [17].

(3) $$\phi_{C}=\frac{\sqrt{N_{1}}}{\sqrt{N_{1}}+\sqrt{N_{2}}}=\frac{1}{1+\sqrt{\frac{N_{2}}{N_{1}}}}$$

**Figure 2:**
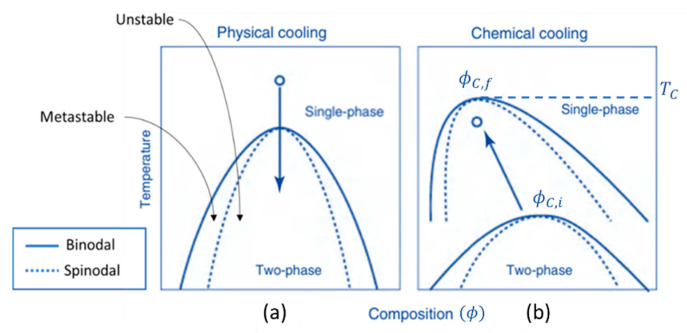
Illustrative phase diagrams of systems with immiscibility window for comparison of physical (**a**) and chemical (**b**) cooling phenomena. Chemical cooling extends the two-phase region by an increased chemical bond, to include the composition initially located in the single-phase region. Reprinted, with adaptations, by permission from Springer Nature Customer Service Centre GmbH: Springer, J. Sol-Gel Sci. Technology, Polymerization-induced phase separation in silica Sol-gel systems containing formamide, Kaji H, Nakanishi K, Soga N, © Kluwer Academic Publishers. Manufactured in The Netherlands (2013) [20].

Concerning spinodal decomposition, in Figure 2a, the arrow pointing down indicates a decrease in the solution temperature and, therefore, a decrease in the degree of freedom between species and subsequently the occurrence of phase separation, physical cooling, or physical quenching. On the other hand, by increasing the number of chemical bonds, such as in a polycondensation process, the degree of freedom also decreases, which makes the mixture behave as if the physical temperature was decreased by reducing the thermal disturbance of the molecular system. This phenomenon, chemical cooling or chemical quenching, is an analogy for the typical physical cooling that exhibits a glass transition [18]. In this sense, Figure 2b shows the effect of the condensation polymerization reaction on course (for instance, the formation of siloxane oligomers), which increases the polymerization degree and moves the critical point $\phi_{C}$ (Equation (3)) in the diagram, leading the system into the two-phase region (chemical cooling or chemical quenching). This latter process was first studied in sol-gel systems (monoliths) by Nakanishi, et al. in the early nineties [18,19,20].

The following expressions explain why the temperature of the critical point ($T_{c}$) is found to rise in the chemical cooling process. The critical interaction parameter $\chi_{c}$ (Equation (4)) is determined by replacing $\phi_{C}$ in the equation of the spinodal $\chi_{s}$. Therefore, $T_{c}$ is inversely proportional to the critical interaction parameter $\chi_{c}$ and directly proportional to $N_{1}$ and $N_{2}$ (Equation (5)) [17].

(4) $$\chi_{c}=\frac{1}{2}{\left(\frac{1}{\sqrt{N_{1}}}+\frac{1}{\sqrt{N_{2}}}\right)}^{2}\;$$

(5) $$\chi_{c}\;≅A+\frac{B}{T_{c}}\;\overset{}{\Leftrightarrow }\;T_{c}=\;\frac{B}{\chi_{c}-A}$$

When polymerization-induced phase separation occurs in a sol-gel system (for instance, a silica system) two phases are present: one in which the condensing alkoxysilanes are present (called the siloxane-rich phase) and another that is low in, or even free of, alkoxysilane content (called the solvent-rich phase). Contrary to the phase separation in standard polymeric systems, the number of statistical segments of the siloxane-rich phase, $N_{2}$, is not static and will increase as the polycondensation reaction proceeds and the silica networks forms, resulting in the decrease of $\phi_{c2}$ and, therefore, in the movement of the binodal and spinodal lines towards the solvent-rich phase (component 1). So, in the case of the system composed by polar solvent (component 1) and siloxanes (component 2), phase separation by spinodal decomposition might only occur, by chemical quench, if $N_{2}$ is high enough ($N_{1}$~1), so that $\phi_{C2}$ is shifted to lower concentrations of component 2 (Equation (3)) and $T_{c}$ goes upward.

## 3. Macroporous Silica (SiO_2_) Monoliths

### 3.1. Pioneering Works

The growth and topology of the macromolecules, which precede gelation in several sol-gel systems, were studied by small-angle X-ray scattering (SAXS) and allowed Schaefer and Keefer in 1984 [21] to conclude that the growth and degree of the cross-linking of polymer chains, occurring during gelation and under catalytic conditions, may in turn induce and control phase separation in silicate sol-gel systems. Such a finding was at the origin of the work by Nakanishi and Soga (in 1991) [19], where, for the first time, the concept of phase separation in sol-gel systems was applied to the macroporosity control in silica-based monolithic materials.

Their work regarded the formation of micrometer range porous morphology in silica gels, prepared from tetramethoxysilane (TMOS)-methanol solutions that contained poly(sodium-4-styrene sulfonate) (NaPSS) as a porogen agent, under acidic conditions using nitric acid [19,22]. NaPSS is a strongly ionic, water soluble polymer, which exhibits a limited solubility in alcohols. Different porosity levels were achieved depending on the NaPSS molecular weight (Mw), NaPSS quantity, and temperature employed in the synthesis, explained by spinodal phase separation, which was induced by the chemical cross-linking and diffusional limitations of the reacting TMOS precursor molecules. In this case, a monophasic solution containing one polymeric compound and polymerizable species was found to experience polymerization-induced spinodal phase separation. The term “chemical cooling” was introduced in this paper [19], since chemical reaction drives the change in free-energy and is used to denote the “induction of spinodal phase separation by chemical reaction”, including that by the polymerization/polycondensation of silicon alkoxides.

NaPSS is an additive component which has a weak interaction with the siloxane network, i.e., hardly establishes hydrogen bonding with the hydroxyl groups on silica surface; the resulting phase-separated system consists of a silica-solvent-rich phase and NaPSS-solvent-rich phase counterpart. This case is an example of the phase separation of the type **“silica versus solvent-polymeric additive”, involving a tri-component system “silica-weakly hydrogen bonding polymer-solvent”.** The phase separation in this system is driven by the entropy loss, due to the polymerization of silica and, thus, depends directly on the mutual Mw of the constituents. Other additives that play a similar role are in situ esterified poly(acrylic acid) (HPAA) [23,24,25,26] and combinations of styrene-divinylbenzene/polydimethylsiloxane), methacrylate/poly(ethylene oxide) (PEO), and acrylamide/PEO [27].

**Figure 3:**
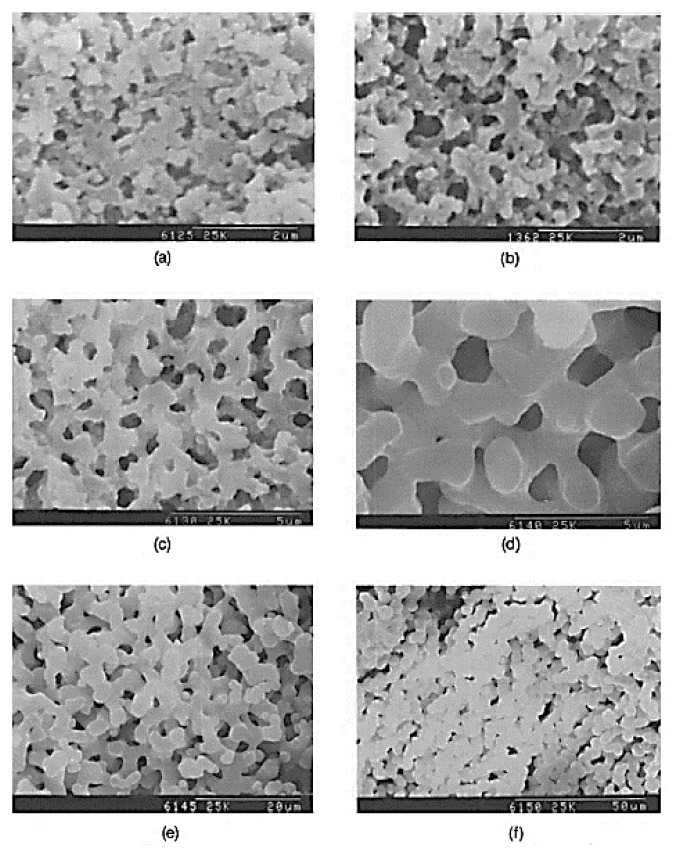
Variations in gel morphology with increasing NaPSS concentration (C, in mol mol^−1^), at 60 °C for NaPSS Mw between 50,000 and 100,000: (**a**) C = 0.179, scale bar = 2 µm, (**b**) C = 0.187, scale bar = 2 µm, (**c**) C = 0.194, scale bar = 5 µm, (**d**) C = 0.201, scale bar = 5 µm, (**e**) C = 0.208, scale bar = 20 μm, and (**f**) C = 0.215, scale bar = 50 µm. Reprinted with permission from K. Nakanishi and N. Soga, J. Am. Ceram. Soc. Copyright © 1991 WILEY-VCH Verlag GmbH & Co. KGaA, Weinheim [19].

As presented in Figure 3, the size of the interconnected structure (characteristic size) increases with the NaPSS concentration, for 60 °C, from ca. 0.2 to 8 µm [19]. The same was also found to happen for 40 °C. The increased temperature promotes the polymerization reactions and improves the mutual solubility of the constituents. The solubility of the NaPSS in the reacting solutions was found to influence the “chemical cooling depth”, i.e., the characteristic size of the porous structure (the higher the solubility, the lower the characteristic size). So, for higher temperatures, more NaPSS is required to achieve the same level of characteristic size.

**Figure 4:**
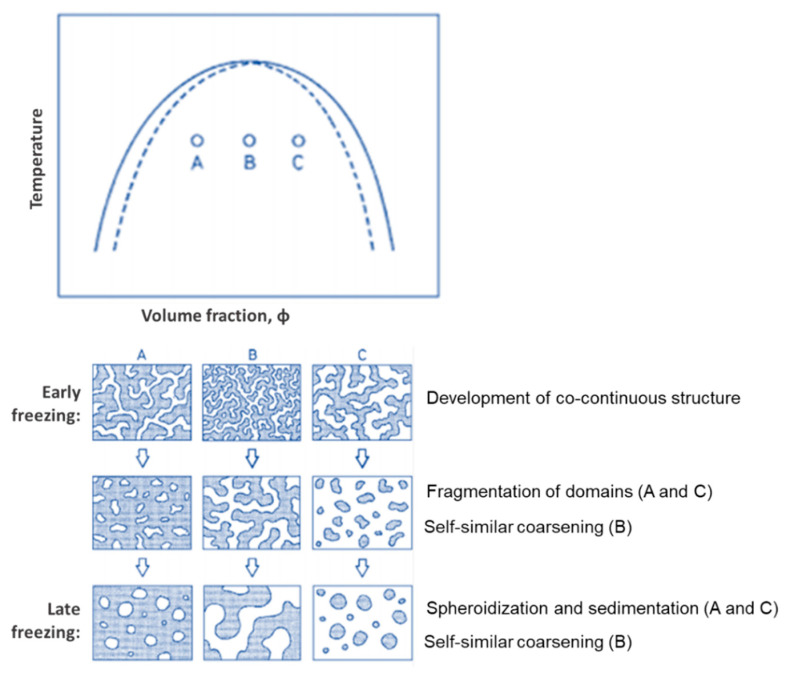
Possible structures at various coarsening stages of spinodal decomposition. Reprinted (and adapted) with permission from K. Nakanishi and N. Soga, J. Am. Ceram. Soc. Copyright © 1991 WILEY-VCH Verlag GmbH & Co. KGaA, Weinheim [19].

Nakanishi and Soga, in their pioneering paper [19], introduced the following mechanistic concept: spinodal phase-separated structure development (upward movement of the phase diagram), followed by the “freezing” of such structures by the subsequent gelation of the silica-rich phase (“chemical cooling” of the system). This results in interconnected morphologies, but various other possible structures with different characteristic sizes can be reached in the coarsening stages of spinodal decomposition (as Figure 4 represents), depending on how far away it is from critical composition, when the structure “freezes”. At off-critical compositions (A and C), the break-up of the interconnected structure (percolation-cluster transition) occurs earlier than at near-critical compositions. Composition B (critical composition, $\phi_{C}$) yields an interconnected macroporous structure independently if it is an early, or late, freezing. An early freezing of the coarsening structure (via gelation) results in a finer structure (small characteristic size) with higher connectivity. The larger the time difference between phase separation and gelation (late freezing), the more coarsened the structure will become (larger characteristic size) and, in some cases (for off-critical compositions), even breaking up into fragmented particles, with no monolithic structure being obtained. If the sol-gel transition occurs at the end of phase separation, an Ostwald ripening structure will be achieved. On the other hand, if it occurs in the initial stage of phase separation, the morphology of such a stage will be obtained.

It is, therefore, concluded that all parameters affecting the relative rates of phase separation versus gelation have a significant influence on the morphology of the final gel, including mesoporosity, degree of macroscopic phase separation, and interconnected macroporosity. Examples of parameters resulting in faster sol-gel transitions are higher temperatures, water/precursor ratio, pH value changes, etc.

The same authors also studied the effects of temperature, Mw, catalytic conditions, and the concentration of carboxylic acid as a polymeric additive on the phase-separated morphology in tetraethyl orthosilicate (TEOS)–HPAA systems [23,24,25,26]. The polymeric additive, in this case, displays the same type of weak interaction with the silica oligomers as NaPSS, but more solubility both in water and alcohols, resulting in different phase separation and gelation behaviors, when compared to the strongly ionic, alcohol insoluble NaPSS. In the TEOS–HPAA system, the interconnected network size was found to depend weakly on the alcohol–water ratio. Experiments where the catalyst (nitric acid):water ratio and reaction temperature were fixed [23] revealed, for increasing quantities of HPAA (with Mw 90,000), an early freezing (lower gelation time). The lowest values of HPAA concentration yielded microporous monoliths without detectable pores by scanning electron microscopy, while, on the other hand, the highest values of HPAA concentration resulted in a fragmented skeleton with spherical silica particles of a few micrometers. Interconnected macroporosity (characteristic of spinodal decomposition) was achieved for intermediate values of HPAA concentration. Regarding co-solvent (ethanol or methanol) concentration, the higher its value, the more diluted the system and the lower the polymerization rate (higher gelation time). Additionally, segregation strength decreases, which drives the system to a later phase separation and results in fine interconnected domains [23].

**Figure 5:**
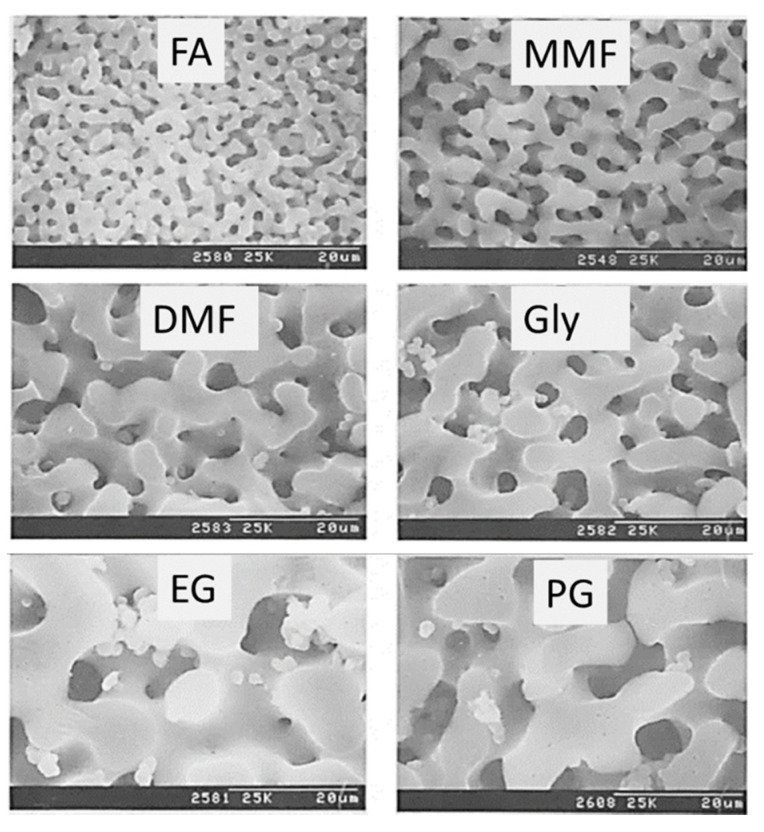
Effect on the gel morphologies with the type of organic solvent for the system TEOS–HPAA (of Mw 250,000) and reaction temperature 80 °C. Scale bar = 20 μm. Solubility parameters, δ_s_ ((cal cm^−3^)^1/2^): formamide (FA)—19.2, N-methylformamide (NMF)—16.1, N,N-dimethylformamide (DMF)—12.1, glycerol (Gly)—16.5, ethylene glycol (EG)—14.6, and propylene glycol (PG)—12.6. Reprinted from J. Non. Cryst. Solids, Vol. 142, N. Soga, K. Nakanishi, Phase separation in silica sol-gel system containing polyacrylic acid. IV. Effect of chemical additives, Pages 45–54, Copyright (1992), with permission from Elsevier [26].

A range of the average Mw of HPAA, between 10,000 and 250,000, was studied [24]. Larger HPAA Mw was reported to lead to a general increase in characteristic sizes and a decrease in gelation time. Nakanishi and Soga [24] also concluded that the amount of HPAA to obtain interconnected gel morphology decreased proportionately to the inverse square root of Mw. On the other hand, a higher concentration of the catalyst (nitric acid) was found to result in greater characteristic sizes [25], as occurred for NaPSS [19]. To conclude this study with HPAA, different chemical additives and organic solvents were tested [26] to assess their influence in the HPAA dissolution, hydrolysis, and gelation processes of the alkoxysilane. As seen in Figure 5, different types of morphologies were obtained, depending on the type of solvent used.

The authors claimed that solvents, which increase the solubility of HPAA (higher solubility parameter) and accelerate the polymerization of silica (e.g., formamide), lead to the formation of fine interconnected structures (smaller characteristic sizes) for a large range of compositions. It was also possible to observe that the structures can be divided into two solvent groups: (i) solvents containing an amide group and (ii) solvents with a polyol functional group (with the former giving finer structures than solvents that contained alcoholic moieties). Sorbitol, one of the sugar-alcohols with a similar molecular structure to glycerol, was also included in this study because of its expected tendency to accelerate the polymerization of silica (by increasing the activity of the acid catalyst). Indeed, it was found to be quite effective in promoting fine domains in the present system, due to its ability to reduce the gelation time and its high compatibility with the other constituents [26].

Following this previous work, Kaji et al. [20], from the same group, further explored the effect of formamide (but without using any organic polymeric additives). This is an example of phase separation of the type **“silica (hydrophobic) versus poor solvent with common solvent”, involving a tri-component system “silica—good solvent—poor solvent”.** This strategy was presented as an alternative to the previous methodology (phase separation between organic polymer-rich and silica-rich domains).

**Figure 6:**
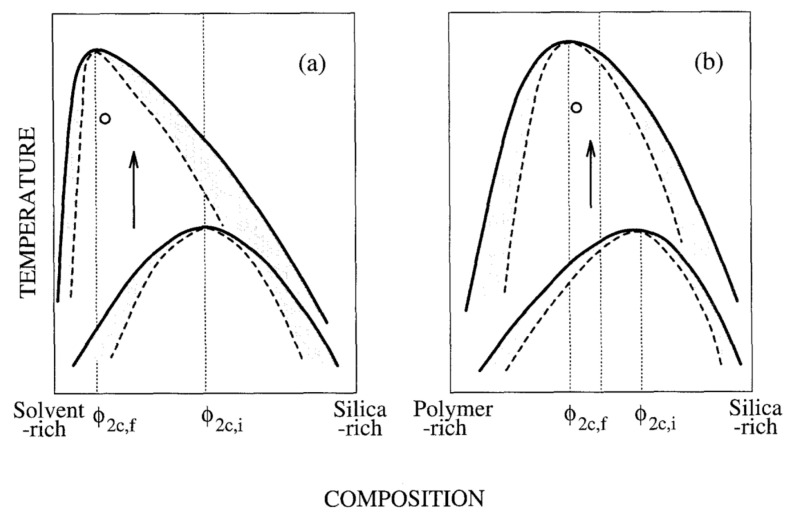
Schematic phase diagrams for the formamide system (**a**) and for an organic polymer containing system (**b**). $\phi_{C,i}$ and $\phi_{C,f}$ are critical compositions just after all the constituents are mixed and during phase separation, respectively. The value of $\phi_{C,f}$ in the formamide system is much smaller (more silica-poor); additionally, binodal and spinodal lines are more asymmetric than those in HPAA system. Reprinted, with adaptations, by permission from Springer Nature Customer Service Centre GmbH: Springer, J. Sol-Gel Sci. Technology, Polymerization-induced phase separation in silica sol-gel systems containing formamide, Kaji H, Nakanishi K, Soga N, © Kluwer Academic Publishers. Manufactured in The Netherlands (2013) [20].

A system consisting of alkoxide (TMOS), a selected amount of water (r = H_2_O/Si = 1.4–1.6), and formamide (e.g., a molar ratio 1:1.5:2.5) was carried out [20], under acid-catalyzed conditions, where formamide acts as a highly polar solvent, i.e., a poor solvent to the polymerizing silica. Formamide is known to exhibit a hydrogen bonding characteristic and is hydrolyzed to produce ammonia, therefore increasing the pH and polycondensation rate in the presence of a strong acid [18]. In this case, the gel phase is composed by silica and a good solvent, and the fluid phase is formed by both the good solvent and the poor solvent (formamide). There is a formation for silica-rich and solvent-rich phases, and so, Equation (3) with $N_{1}$(solvent) = 1 can be used in the formamide system, contrary to the case of HPAA system, where $N_{1}$(organic polymer+solvent) is much larger than unity. This results in a $\phi_{C,f}$ shifted to a lower silica concentration during phase separation for the formamide system, as well as more asymmetric binodal and spinodal lines, as shown in Figure 6 [20].

Such qualitative explanation, based on the effect of the polymerization degree on the Flory-Huggins’ type $\Delta G_{m}$, leads to the observation that the composition region in the formamide system that gives interconnected structures tends to be more silica-poor than that of the HPAA system, resulting in the formation of continuous, interconnected, (but thinner) silica skeletons. In fact, it was also reported [20] that the composition region that gave interconnected structures (in the formamide system) was much more limited and their characteristic sizes were much smaller, typically lower than 10 µm, than those of the previously reported systems [13,17,18,19,20] containing an organic polymer.

The influence of the Si precursor on the gelling process and, therefore, on the gel morphology was also reported [18]. Methyltrimethoxysilane (MTMS), when compared with tetramethoxy silane (TMOS), exhibits reduced functionality, which, together with the presence of unhydrolyzable methyl groups attached to silicon, results in a slower gelling process (i.e., a slower structure formation, giving more time for the coarsening of the domains). It could lead to a delayed polycondensation versus phase separation; however, the dependence of gel morphology on reaction parameters might be easier to control.

These pioneering studies were followed by experiments incorporating additives to induce phase separation on the polymerization of silica, which displays a strong interaction with silanols through hydrogen bonding or electrostatic attraction. These additive molecules expose their hydrophobic moieties towards surrounding polar solvents and “consume” the surface hydroxyl groups of the hydrolyzed silanes, reducing the affinity with the polar solvent. Thus, in this case, phase separation takes place between the solvent phase and the Si oligomers covered with the additive molecules. This is an example of phase separation of the **silica–polymeric additive versus solvent, involving a tri-component system “silica–strongly hydrogen bonding polymer–solvent”.** This category of phase separation, driven by repulsive interactions between the solvent mixture and the polymeric additive adsorbed on the Si oligomers, is an enthalpy-driven process.

The minimum requirement for the polymeric additive component to induce such kinds of phase separation is a strong hydrogen bond with the Si oligomers, which results in a lowered affinity with the polar solvent phase. Studies show that the polymeric additive’s Mw does not play a great role in this process and that polymerizing silica, despite being able to distribute both at the polymeric additive-rich and at the solvent-rich phases, always gels prior at the polymeric additive-rich phase [18]. This indicates that siloxane oligomers present at the polymeric additive-rich phase have higher Mw, or/and are located closer to each other, than those in the solvent-rich phase. A compromise, in terms of hydrogen bond strength, must be taken into account in order to promote phase separation without jeopardizing polycondensation reactions between silanols. Examples of polymers (or other compounds fulfilling this requirement) are PEO, poly(acrylamide) (PAAm), poly(vinylpyrrolidone) (PVP), nonionic surfactants containing polyoxyethylene units [28], and ionic surfactants (such as sodium alkylsulfates and alkyltrimethylammonium salts) [27].

**Figure 7:**
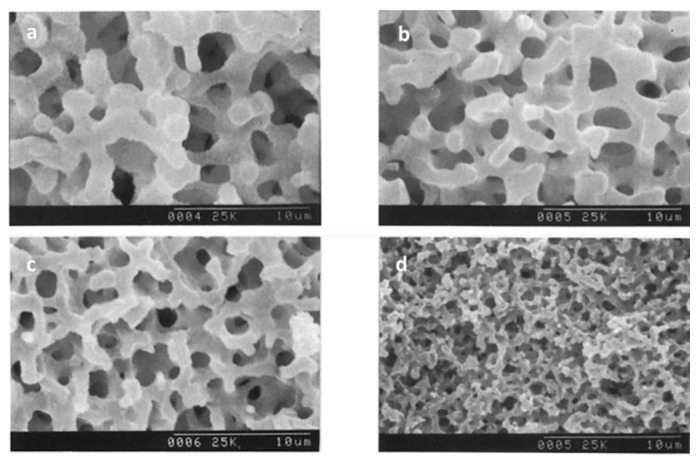
Scanning electron photomicrographs of dried gel morphology for increasing quantities of PEO (100,000 Mw), in a synthesis that involves 6.51 g of TEOS, 0.81 g f 60% aq. HNO_3_, and 8 g of H_2_O: (**a**) 0.30 g PEO; (**b**) 0.40 g PEO; (**c**) 0.50 g PEO; (**d**) 0.60 g PEO. Scale bar = 10 μm. Reprinted by permission from Springer Nature Customer Service Centre GmbH: Springer, J. Sol-Gel Sci. Technology, Phase separation kinetics in silica sol-gel system containing polyethylene oxide. I. Initial stage, Nakanishi K, Yamasaki Y, Kaji H, Soga N, Inoue T, Nemoto N, © 1994 Kluwer Academic Publishers, Boston. Manufactured in The Netherlandss (1994) [29].

One of the first polymeric additives to be tested in this type of phase separation system was PEO, which is a non-ionic polymer that exhibits a LCST-type phase diagram with water, i.e., dissolves homogeneously in water at room temperature but phase separates upon heating to near the boiling point of water. PEO molecules interact with silica oligomers via the hydrogen bonding between the ether group in PEO and the silanol group in silica. Phase separation studies in silica sol-gel systems containing PEO, performed by the same research group [29,30,31], showed that the volume fraction of pores mainly depended on the solvent fraction, and the domain’s size decreased with an increase of the PEO/silica ratio, as observed in Figure 7.

Gelation becomes slower (longer gelling time, t_g_) with an increased amount of PEO because the strong interaction of silica with PEO reduces the available silanol sites for polycondensation. For instance, t_g_ increases from 103 to 123 min (from a to d) in Figure 7 [29]. Moreover, the domain size of the solvent-rich phase generally decreases with the increase of PEO at a relatively high concentration range, contrary to the case of TMOS-NaPSS and TEOS–HPAA systems (entropy-driven) previously reported. The effects of the Mw of PEO and the reaction temperature were also investigated. Nitric acid was employed as a catalyst for hydrolysis, and PEO grades of 8300, 10,000, and 100,000 Mw were used. The overall phase separation tendency of the PEO-silica-solvent system was consistently interpreted by considering the lowered solubility of PEO that was partly adsorbed onto silica oligomers in an aqueous solvent mixture. Therefore, the higher the Mw of PEO, the lower the concentration at which phase separation was found to occur. The present PEO-silica-solvent ternary system was reported to display a typical UCST behavior: the increased temperature leads to a delay of the onset of phase separation and accelerates the gel formation [31], therefore leading to smaller interconnected domains.

The incorporation of several kinds of nonionic surfactant additives, namely polyoxyethylene nonylphenylethers, from 1.2–2.2 g to 5.15 g of TMOS [28], also resulted in well-defined interconnected macropores, while the mesopores formation was tailored by solvent exchange procedures, either in HNO_3_ solution or NaOH solution. The length of the oxyethylene units in the surfactant molecules was found to play a role in the mesopore characteristics, in the sense that the shorter oxyethylene chains tended to be more incorporated in the gel phase and gave higher mesopore volume.

The selected works, referenced in this sub-section and dated from the decade of 1990–2000, are the most representative studies reported on the obtention of silica porous monoliths, involving the three different sol-gel/phase separation systems: (a) silica—weakly hydrogen bonding polymer—solvent; (b) silica—good solvent—poor solvent; (c) silica—strongly hydrogen bonding polymer—solvent.

### 3.2. Further Developments on Inorganic and Inorganic-Organic SiO_2_ Monoliths

Tetra-functional silanes, such as TEOS and TMOS, were the main Si precursors employed in the early works regarding this topic. Their relatively slow kinetics makes these compounds very easy to handle and, thus, very attractive. However, silanes with organic functionalities have been used to transfer other functionalities to the resulting inorganic–organic hybrid organosiloxane monoliths. For instance, the presence of alkyl groups covalently attached to Si atom, such as in MTMS, dimethoxydimethylsilane (DMDMS), octyltriethoxysilane (nOTES), or hexadecyltrimethoxysilane (HDTMS) makes the siloxane oligomers more hydrophobic and, therefore, more prone to phase separation, when compared to tetra-functional silane systems. It should be stressed, as well, that the presence of specific organic functionalities, such as glycidyloxy, contributes to polymerization reactions, which also might favor phase separation processes [2] and might avoid the use of phase separation inducer additives, such as those referred in Section 3.1. Additionally, hybrid compositions, e.g., those resulting from MTMS and DMDMS, result in more flexible xerogels. Moreover, phenyltrimethoxysilane (PhTMS) has been used as a precursor to prepare phenyl-modified macro/mesoporous hybrid silica monoliths, targeting mechanical resistance, better pH stability, and resistance to swelling and shrinking in many solvents [32].

Nonionic surfactants have been employed either to suppress excessive phase separation in hybrid compositions, and to tailor mesoporosity giving bimodal hierarchical pore morphologies.

Still on inorganic SiO_2_ gels, Nakanishi et al. [28], in 1998, and Sato et al. [33], in 2001, reported the multiscale templating in TMOS derived systems, using nonionic surfactants, polyoxyethylene nonylphenylethers and PEO-b-PPO-b-PEO triblock copolymers, respectively. The latter one was Pluronic P123, having the molecular formula of (EO)_20_(PO)_70_(EO)_20_. Later on, P123, and another triblock copolymer, F127, of molecular formula (EO)_106_(PO)_70_(EO)_106_, were used in combination with trimethyl benzene (TMB) cosolvent or swelling agent [34]. These surfactants functioned as structure-directing agents and phase separation inducers, whereas TMB worked as a pore expander and enhanced the interaction among the hydrophobic moieties of the structure-directing agent. Studying the relationship between the polymerization-induced phase separation, the cooperative assembly of the surfactant micelles and silica oligomers and the sol-gel transition was a central part of the work. Higher amounts of TMB coarsened the initially isotropic macroframeworks, giving a fibrous appearance. The shape of the pores changed from cylindrical to spherical for increasing concentrations of TMB. These nonionic surfactants are known to make hydrogen bonding between the silanol groups (Si-OH) and the ether oxygens of EO units. Cationic surfactants, such as n-hexadecyltrimethylammonium salts (CTAB for bromide or CTAC for chloride), on the other hand, have also been used for generating phase separation and are known to interact with the silica domain by orienting the polar head groups toward silica. The ionic attractive interaction and hydrophobicity of the attached alkyl group were concluded to cooperatively determine the phase separation tendency [35]. In these former cases, the amphiphilic block copolymer or cationic surfactants were used solely as phase separation inducers and supramolecular templating agents; however, the mixture of C_14_TAB and C_18_TAB with PEO was also explored by Smått et al. [36]. Structures of high order, such as hexagonal or cubic arrays of cylindrical pores, could not be evidenced; however, the use of triblock co-polymers proved to be effective in templating the mesopores in the resulting macroporous silica gels.

Sol-gel/phase separation-derived, alkylene-bridged polysilsesquioxanes, with macroporosity, were obtained by Nakanishi et al., from a bis(trimethoxysilyl)hexane (BTMH)–water–methanol system in acidic conditions [37] and compared to a MTES derived monolith. No polymeric phase-inducer additive was used in this work, nor formamide, or other gelation promoter. The presence of unhydrolyzable methyl group attached to silicon, in MTES or MTMS silanes, was found to extend the composition region of phase-separated morphology, even to the water-silane binary compositions [18]. In the case of alkyltrialkoxysilanes, the phase separation tendency increases rapidly with an increase of the length of alkyl chain directly bonded to Si atom. However, in the case BTMH system, the co-continuous macroporous structure was obtained in the composition region containing higher water concentration (Si/water~1/30, molar ratio), than with MTES. Combinations of 4- and 3-functional alkoxysilanes, namely TMOS and vinyltrimethoxysilane (VTMS), and the employment of formamide as an additive polar solvent, resulted in the occurrence of phase separation in extended composition regions with high water concentration, especially with larger amounts of VTMS (e.g., TMOS/VTMS = 20/80 molar ratio) [38]. The domain size and pore volume of the macroporous gels could be controlled by the alkoxide:water ratio and total solvent fraction, respectively.

**Figure 8:**
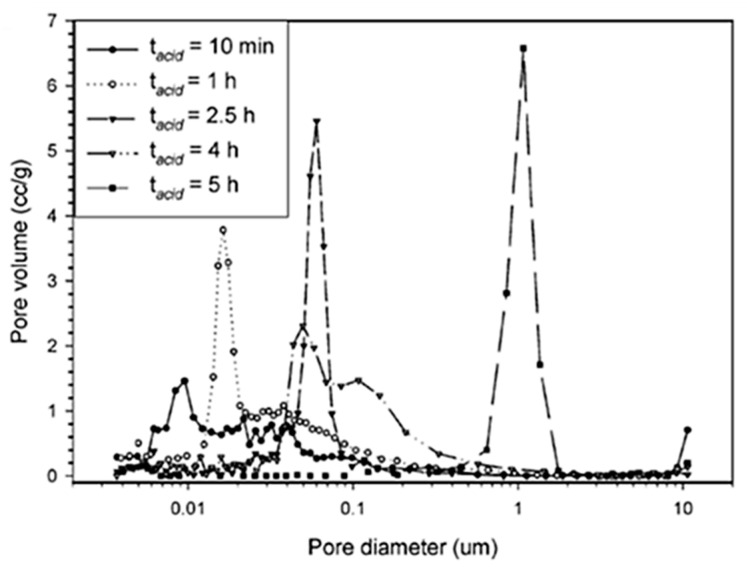
Differential pore size distribution of PMSQ monoliths achieved by using different durations of the acidic step, from 10 min to 5 h (dried at 300 °C). Measurements carried out by mercury porosimetry. Reprinted (and adapted) with permission from Dong H, Brennan JD (2006) Controlling the morphology of methylsilsesquioxane monoliths using a two-step processing method. Chem Mater 18:541–546. https://doi.org/10.1021/cm051900n [39]. Copyright 2006 American Chemical Society.

In 2006, Dong et al. [39] presented an acid/base two-step method to obtain macroporous polymethylsilsesquioxane (PMSQ) monoliths from MTMS, without the use of phase separation inducer additives. In this study, hydrolysis and polycondensation proceed in acidic and basic aqueous media, respectively. Systems in which the gelation time was only slightly longer than that of the phase separation time (in 4–10 min) showed a significant fraction of macropores. Therefore, it was claimed that increasing the duration of the acid step, the size of polymerized clusters increases, leading to an early onset of phase separation, relative to gelation and, therefore, to larger macropore size, with narrow distribution [39], as observed in Figure 8. The observed shift from mesopores to macropores resulted in a decrease of surface area from 426.4 to 59.3 m^2^/g.

A few years later, Kanamori et al. studied the transition of mesoporous to hierarchical macro/mesoporous gels in a hybrid PMSQ system [40] derived from MTMS, and Tao et al. reported the application of these new hierarchical monoliths to oil removal [41] in 2011. An acid/base two-step method (one-pot reaction) was employed, together with the use of a triblock copolymer, F127, which is known to establish attractive interactions (weak) with methylsiloxane networks. It was concluded that mesopores are formed through aggregation of PMSQ colloids, while macropores, which result from controlled phase separation by spinodal decomposition, were due to the presence of F127 when used at very low quantities. It should be stressed, however, that for larger amounts of F127 (above 0.34 g) macropores were found to decrease in size. Surfactant was extracted by solvent exchange processes.

Kurahashi et al. [42] reported the effect of a variety of nonionic PEO-b-PPO-b-PEO triblock copolymers with different Mw and PO/EO ratios (HLB value) on the phase separation behavior of a MTMS-based system. Most of the surfactants were found to suppress phase separation, except surfactants L35 (low Mw) and P123 (very high hydrophobicity, high PO/EO ratio), which were the only ones to generate opaque gels, an evidence of successful phase separation, for the range of concentrations studied. Another work suggests that these surfactants, when displaying shorter oxyethylene chains, tend to be incorporated more in the gel phase, giving higher mesopore volume [28].

**Figure 9:**
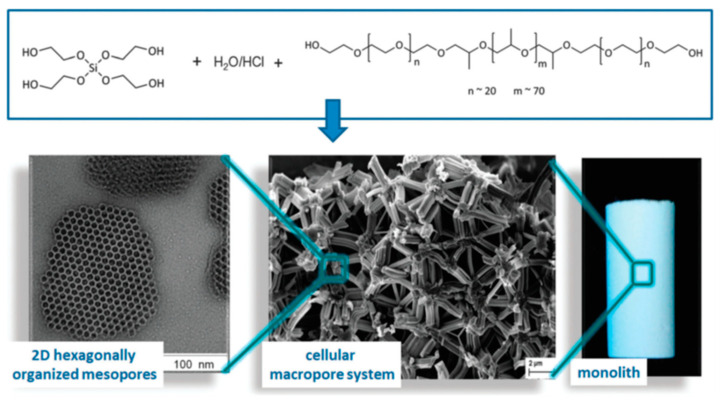
Increasing magnification, from right to left, of a silica monolith prepared from glycol-modified silane in the presence of P123 at pH = 1. Republished with permission of Royal Society of Chemistry (Great Britain), from Sol-gel synthesis of monolithic materials with hierarchical porosity, A. Feinle, MS. Elsaesser, N. Hüsing, 45, 12, 2015 [1]; permission conveyed through Copyright Clearance Center, Inc.

The hydrolysis of alkoxysilanes leads to the release of low-Mw alcohols, which might negatively impact the supramolecular arrangement of block copolymer surfactants. One solution is their removal by vacuum distillation. Additionally, Hüsing et al. [43], in 2003, created a synthetic route to achieve an alternative silica source, a glycol-based silane, tetrakis(2-hydroxyethyl)orthosilicate, designed to not release those low-Mw alcohols, but instead, ethylene glycol. Monoliths containing pore sizes on different length scales (from micro to macropores) were generated by employing a variety of hydrophilic diol- and polyol-modified silanes [44], and P123 as a template, represented in Figure 9, which was posteriorly extracted using supercritical carbon dioxide.

The addition of inorganic salts, such as NaCl, Na_NO3_, or Na_2_SO_4_ [45] was another methodology reported to promote the formation of ordered mesostructures in macro/mesoporous hierarchical SiO_2_ monoliths, when a weak acidic medium and surfactant P123 are used. The cross-linking degree of the SiO_2_ monolith was found to be higher in the presence of these inorganic salts (disappearance of *Q*^2^ site and increased ratio of *Q*^4^/*Q*^3^ in the ^29^Si MAS NMR spectra), due to the strong ionizing capacity of the anions $Cl^{-}$, $NO_{3}^{-}$, and $SO_{4}^{2-}$ that increases the hydrolysis and condensation rate of TMOS.

Table 1 lists a series of synthesis strategies to achieve hierarchically structured SiO_2_ mo_no_liths with macro and mesopores, via sol-gel/phase separation.

The applications of silica-based monoliths can be extended to nontraditional fields, such as photocatalysis. As an example, SiO_2_ porous monoliths prepared in a similar fashion as in [36], have been subjected to vacuum impregnation of sodium tungstate to prepare porous WO_3_/SiO_2_ monoliths. They exhibited a narrow band gap (2.5 eV) and are suitable for visible light photocatalysis targeting the degradation of toxic pollutants to reduce water pollution [46].

## 4. Macroporous SiO_2_-Based Multicomponent Oxide Monoliths

Porous materials with extended chemical compositions are highly desirable for greater chemical range and variety. The first developments in pure SiO_2_ macroporous monoliths were soon followed by the synthesis of multi-oxide, silica-based compositions using the co-gelation method with other metal oxides. However, the reactivity and stereochemistry of the latter ones is different from those of silicon alkoxides, so synthesis protocols must be adapted to avoid the precipitation of the highly reactive metal alkoxides. Common strategies include low synthesis temperatures and the employment of chelating ligands.

As far as we are concerned, the first macroporous multi-oxide monolith synthesis through a sol-gel/phase separation was published by Nakanishi et al. in 1992 [47], for the SiO_2_-TiO_2_ system, which is known by the ultra-low expansion properties. It involved the hydrolysis and condensation of the precursors TMOS and titanium tetrabutoxide (TBOT), under strong acidic media and HPAA as a phase separation inducer, as in previous works for SiO_2_ composition [23,24,25,26]. Lower hydrolysis temperatures had to be used, in this case, to control the high reactivity of TBOT, which was found to modify the polycondensation mechanism, in a similar fashion as base-catalyzed systems and retard the occurrence of phase separation. It should be stressed that only ca. half amount of the initially added titanium was incorporated in the gel network.

The use of PEO as a phase separation inducer has proved to be advantageous for controlling the macroporous morphology. Macroporous SiO_2_-TiO_2_ monoliths using this strategy (PEO, Mw 20,000) were reported in 2012 by Ruzimuradov et al. [48], where the influence of various titanium precursors (titanium isopropoxide, TiPOT, TBOT, titanium tetrachloride (TiCl_4_), and 30% titanium sulfate solution (Ti(SO_4_)_2_)) on phase separation tendency was investigated, for systems containing 5 and 7.6 wt% TiO_2_. In the two-step pre-hydrolysis method, TEOS was first partly hydrolyzed under acidic media before the addition of the TiPOT precursor while using a cosolvent and a limited quantity of water. TiPOT only condensates with the few available -OH moieties and when reactivity is controlled. In the acetylacetone (acac)-complexation route, TEOS, TiPOT, and acac were mixed, and then an acidic aqueous solution containing PEO was added.

Titanium alkoxides were found to enable SiO_2_-TiO_2_ monoliths with high Ti incorporation when acac was used as a chelating agent, but largely decreased phase separation tendency. In this case, a low temperature reaction was necessary for macropore formation. On the other hand, titanium salts (titanium chloride and titanium sulfate) showed a small effect on phase separation tendency and yielded, as well, SiO_2_-TiO_2_ monoliths with high Ti dispersion, for the studied compositions.

Yang et al. [49], in 2013, addressed the challenge to fabricate ordered mesoporous channels in the SiO_2_-TiO_2_ bicontinuous macroporous framework walls. They were able to synthesize hierarchical SiO_2_-TiO_2_ monoliths with higher Ti contents, by a double-template technique, involving water soluble organic polymers (PEO 10,000 Mw) and triblock copolymers. The addition of fluoride (NH_4_F) promoted the incorporation of Ti atoms in the silica framework.

Table 2 compiles different synthesis strategies, based on co-gelation, for a variety of binary and other multicomponent oxide silica-containing hierarchically porous monoliths.

Examples of other binary silicate systems with high SiO_2_ content have already been reported, such as SiO_2_-ZrO_2_ [50,62,63] with ZrO_2_ contents up to 21.3 wt% and SiO_2_-Al_2_O_3_ sol-gel systems [51,53,54]. An ice bath was used to limit the reactivity of the metal alkoxides. The addition of Zr alkoxide causes the increase of condensation rates and limits the coarsening of domains formed by spinodal decomposition. This effect, however, could be compensated by decreasing the polarity of the solvent, i.e., increasing the length of the alkyl chain of the alcohols (from methanol to 2-propanol) [62]. The incorporation of ZrO_2_ into the SiO_2_ structure increased thermal resistance, since the SiO_2_–ZrO_2_ gel maintained interconnected morphology up to 1200 °C, while the pure SiO_2_ gel maintained it up to 1000 °C [63]. Regarding SiO_2_-Al_2_O_3_ monoliths, Takahashi et al. (2001) and Murai et al. (2004) reported the synthesis of such monoliths using PEO as a template [51,52] and found that the macropore size could be adjusted by altering the starting compositions. Wu et al. (2007) and Yang et al. (2010) further elucidated on the acidic properties and tailored the mesoporous structure of such mixed oxides [53,54], by adding, as well, nonionic surfactants, namely polyoxyethylene cetyl ether containing 10 oxyethylene units (C_16_EO_10_) or P123 as structure-directing agents. The Al alkoxide precursor was found to lead to a relatively higher Al content in the SiO_2_-Al_2_O_3_ monoliths than the Al nitrate precursor.

The epoxide-assisted sol-gel processing starting from metal–salts has proven to be useful in achieving highly homogeneous binary oxides and was firstly implemented by Guo et al. (2013) for SiO_2_-Al_2_O_3_ systems, using PEO as a phase separation inducer [55]. The reported homogeneity for the resulting materials is mainly due to the stable and uniform dispersion of metal cations in the starting solution. Epoxides, such as propylene oxide (PO), are protonated by an acid (H^+^), and an irreversible ring-opening reaction is made to occur by a nucleophilic anionic base. The use of PO enables a uniform and rapid raise of pH, which promotes hydrolysis and polycondensation. In this work [55], formation of a crystalline phase (mullite) occurred, by heat treatment at 1400 °C and, despite not impacting the macroporous morphology of the monoliths, the same did not happen to meso or nanopores, since it led to a drastic decrease of the surface area, from 417 m^2^/g to 5.4 m^2^/g.

Experiments involving the addition of nickel into the silica-PEO system [56] showed that its incorporation had a negligible effect on the morphology formation of the porous monoliths, regardless of the loading process employed, namely co-gelation, solution exchange, or impregnation. However, NiO dispersion was found to vary: Ni was selectively incorporated into the gel framework, prepared by co-gelation, as fine particles in the system with PEO, probably due to a possible interaction of PEO, both with silica and Ni cations, while the latter two methods resulted in Ni aggregation in the macropores. Samarium ions (Sm^2+^) were also incorporated within the silica and silica-alumina matrices of macroporous monoliths, using SmCl_3_·6H_2_O and thermal reduction of Sm^3+^ to Sm^2+^ [64].

In 2006, the co-gelation method was adapted to produce bimodal porous copper-silica and nickel-silica monoliths by using nitrate precursors, by Zheng et al. [57]. Similarly to [56], it was concluded that PEO interacted with both silica and nickel cations, but not significantly with copper cations. These latter ones tended to aggregate as copper salts in the drying step of the wet gel and decomposed into CuO particles by heating.

**Figure 10:**
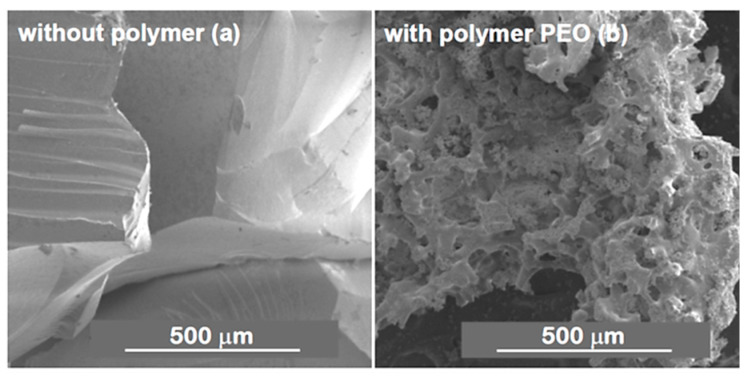
SEM photomicrographs of heat-treated monoliths of composition 70%SiO_2_–30%CaO (mol%), prepared by (**a**) the sol-gel technique without addition of PEO and (**b**) the sol-gel/phase separation technique with addition of PEO. Reprinted by permission from Springer: Springer Nature, Journal of Materials Research, Sol-gel-derived glass scaffold with high pore interconnectivity and enhanced bioactivity, A.C. Marques, R.M. Almeida, A. Thiema, S. Wang, M.M. Falk and H. Jain [58]. Copyright Springer Nature 2009.

**Figure 11:**
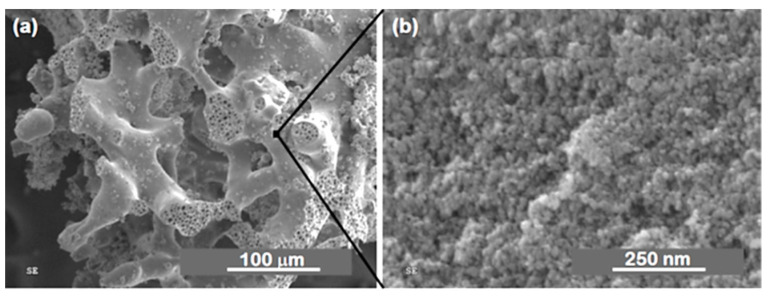
SEM photomicrographs of a heat-treated monolith of composition 70%SiO_2_–30%CaO (mol%), prepared by the sol-gel/phase separation technique with the addition of PEO: (**a**) magnification of 300× and (**b**) magnification of 120,000×. Reprinted by permission from Springer: Springer Nature, Journal of Materials Research, Sol-gel-derived glass scaffold with high pore interconnectivity and enhanced bioactivity, A.C. Marques, R.M. Almeida, A. Thiema, S. Wang, M.M. Falk and H. Jain [58]. Copyright Springer Nature 2009.

Marques et al. [58,59,65] reported on resorbable glass scaffolds of molar compositions 70%SiO_2_–30%CaO and 77%SiO_2_–19%CaO–4%P_2_O_5_, using several different strategies for the synthesis, as shown in Table 2. The obtained monoliths exhibited high pore interconnectivity, enhanced bioactivity, and consisted of macropores of ~5 to 300 µm in size within a coral-like gel/glass skeleton, and nanopores ~5–20 nm in size. PEO (PEO/TMOS ~10 wt%), or P123, was employed for achieving such macroporosity, by phase separation. Deionized (DI) water and hydrofluoric acid were added before the gelation step. Removal of the solvent-rich phase by evaporation and polymer burn-off introduces macroporosity. Figure 10 shows SEM images of heat-treated 70%SiO_2_–30%CaO monoliths prepared (a) without the addition of PEO and (b) with the addition of PEO, revealing an extensive macroporosity in the latter case, also shown in Figure 11a, where large pore necks are observed. The size of nanopores was found to increase with the calcium and phosphorous content and with solvent exchange procedures (using DI water and ammonia), as well; whereas, macropores remain unaffected [65].

**Figure 12:**
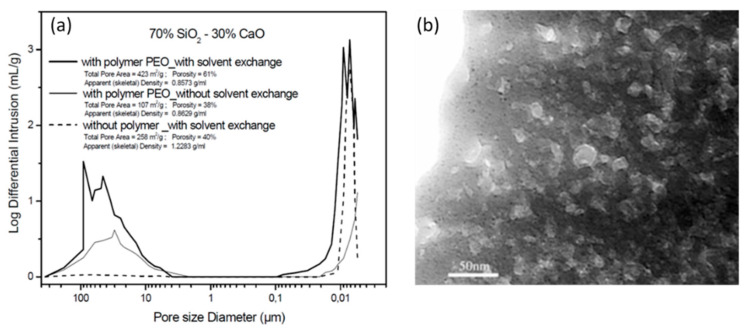
(**a**) Interconnected pore size distributions for SiO_2_-CaO heat treated monoliths, determined by Hg intrusion porosimetry. The samples analyzed were prepared both with and without PEO, as well as with and without solvent exchange. Reprinted by permission from Springer:Springer Nature, Journal of Materials Research, Sol-gel-derived glass scaffold with high pore interconnectivity and enhanced bioactivity, A.C. Marques, R.M. Almeida, A. Thiema, S. Wang, M.M. Falk and H. Jain [58]. Copyright Springer Nature 2009; (**b**) Bright field TEM photomicrograph of the monolith prepared with PEO and solvent exchange procedure. Reprinted by permission from Springer:Springer Nature, Journal of Sol-Gel Science and Technology, Nano/macroporous monolithic scaffolds prepared by the sol-gel method, A.C. Marques, H. Jain, C. Kiely, K. Song, C.J. Kiely and R.M. Almeida [65]. Copyright Springer Nature 2009.

Figure 12a shows the pore size distribution curves obtained from Hg porosimetry. The peak macropore size for the monoliths prepared with PEO was around 60 µm and ranged from ~5 to 300 µm, whereas the sample prepared without PEO did not present any measurable amount of macropores. Mesopores were found to be larger, ~10 nm in size for samples prepared with solvent exchange, than without solvent exchange. The SEM micrograph (Figure 11b) shows the presence of mesopores, and the TEM micrograph (Figure 12b) shows that their size varies between ca. 10 and 20 nm. X-ray ultramicrographs were also acquired and corroborate these findings [65].

Regarding ternary systems, cordierite (MgO–Al_2_O_3_–SiO_2_) and Al_2_O_3_-SiO_2_-TiO_2_ porous monoliths were developed by Guo et al. (in 2014) and Sun et al. (in 2016), respectively [60,61]. Porous cordierite monoliths were prepared via the sol-gel process in the presence of a PAAm phase separation inducer [60]. This ternary system has numerous applications because of its excellent mechanical properties, such as thermal shock resistance and good chemical durability. Again, PO was used to act as an acid scavenger, mediating the gelation through the ring-opening reaction. Magnesium chloride, aluminum chloride, and TMOS were used as precursors. Regarding the Al_2_O_3_-SiO_2_-TiO_2_ ternary system, PEO and formamide were used as a phase separation inducer and gelation agent, respectively, and acetic acid was used as a chelating agent [61]. Stirring was done under ice-cooling conditions. The obtained amorphous gel was transformed into Al_4_Ti_2_SiO_12_ after a heat treatment at 1050 °C, losing its meso/micropores due to crystallization and sintering, but keeping the macroporosity, as was already evidenced in other compositions.

## 5. Macroporous Non-Siliceous Single Oxide, Multi-Oxide, and Non-Oxide Monoliths

Single- and multi-oxide monoliths with 3D interconnected macropores, sometimes with mesoporosity (hierarchical), can be prepared by the transient state’s spatial arrestment during the spinodal decomposition caused by the gelation and/or self-assembly of the precursors. By controlling the reactional parameters, e.g., types of precursors, precursor/water molar ratio, pH, temperature, presence of cosolvents, gelling agents, and phase separation inducer additives, the morphology of the 3D macropore network can be controlled. Varied synthesis strategies have been pursued for different types of non-siliceous materials (oxides and non-oxides), including recent developments on carbon (C), C/oxides, and metal organic frameworks (MOFs).

The study of silica-based multi-oxide monoliths established the baseline for the study of non-siliceous single- and multi-oxide systems. As far as we are concerned, the first non-siliceous porous monoliths prepared by sol-gel/phase separation were made of TiO_2_ [66]. However, structural development control during hydrolysis and polycondensation is harder to manage than it is for silica-based materials, due to the high reactivity of titanium alkoxides. The electronegativity of the central metallic atom plays an important role in hydrolysis and condensation rates. The lower electronegativity causes them to be more electrophilic and, therefore, less stable to nucleophilic reactions, such as hydrolysis and condensations. Because of the greater reactivities and rapid kinetics, it is essential to have greater control of hydrolysis’ conditions, including moisture, to avoid obtaining precipitates.

### 5.1. Titania Macroporous Monoliths

It is common knowledge that titanium dioxide monoliths are very important, for instance, in the field of catalysis, among other applications. TiO_2_ monoliths achieved by polymerization-induced phase separation were produced through two approaches: 1—formamide-based system [66,67,68] and 2—chelated system [69,70].

#### 5.1.1. Formamide-Based Systems

The first approach to achieve macroporous TiO_2_ monoliths involved the use of aqueous titania colloid, instead of highly reactive titanium alkoxide [66], by Fujita et al. in 2004. The gelation/assembly (physical aggregation) of titania (anatase) colloidal particles was controlled, in this case, by the intrinsic and gradual increase of pH, due to the hydrolysis of formamide in acidic conditions that produces ammonia. An increase in pH leads to a reduction in zeta potential (low-charged surfaces), weak repulsive forces, and, therefore, a higher tendency for the colloidal particles´ agglomeration. In parallel, phase separation was induced by the presence of PEO in the reaction mixture. Basically, the thermodynamic instability was generated by the repulsive interaction between the solvent and the PEO chains adsorbed on the aggregating TiO_2_ particles. Since these wet gels are fragile, a freeze-drying process was needed to maintain the monolithic gel. It was also concluded that during the gelation process, the small primary particles aggregate into larger secondary particles, resulting in a wide range of mesopores, and specific surface areas of 350 m^2^/g.

In 2006, Konishi et al. [68] reported a method for synthesizing porous TiO_2_ monoliths using an alkoxy-derived sol-gel process accompanied by phase separation. This method involved a relatively high concentration of acid solution (HCl) to suppress the condensation of hydrolyzed Ti alkoxide by electrostatic repulsion, a gelation agent (formamide), and no polymeric additive as a phase separation inducer (template-free). Basically, this process was a one-pot synthesis with an initial hydrolysis step under a strong acidic condition and a subsequent condensation step under alkaline condition, from the formamide decomposition into ammonia. In this sense, polycondensation is favored, inhibiting heterogeneous TiO_2_ precipitation. Another advantage of formamide is its high polarity and hydrogen bonding character, contributing to phase separation. There is no need for strong hydrogen bonding additives, such as PEO. A comparison with colloid derived TiO_2_ gels was carried out in this paper. However, it should be stressed that the search for milder sol-gel reactions (especially in an industrial environment), led to the development of new methods, involving chelating agents and mineral salts.

**Figure 13:**
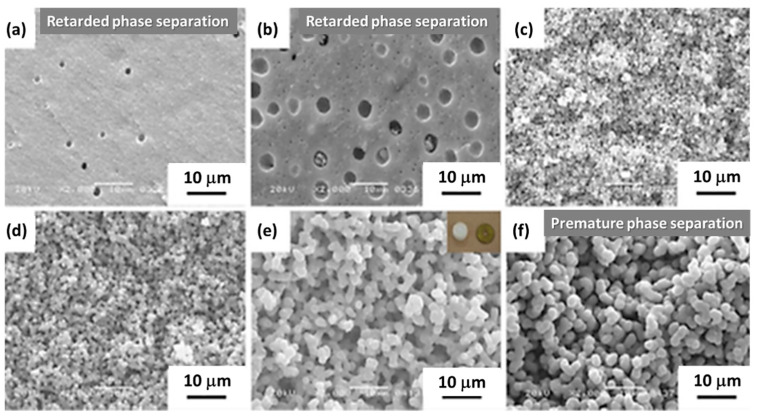
SEM photomicrographs of the dried TiO_2_ gels prepared with different PVP contents (x): (**a**) 50 mg; (**b**) 70 mg; (**c**) 90 mg; (**d**) 100 mg; (**e**) 110 mg; (**f**) 115 mg (quantities used in the formulation: 0.5 g TiOSO_4_·xH_2_O: 1 mL H_2_O: 0.3 mL EG: 0.248 mL formamide: x mg PVP). Reprinted by permission from Springer Nature Customer Service Centre GmbH: Springer, J. Sol-Gel Sci. Technology, Sol-gel synthesis of macroporous TiO_2_ from ionic precursors via phase separation route, Li W, Guo X, Zhu Y, Hui Y, Kanamori K, Nakanishi K, © Kluwer Academic Publishers. Manufactured in The Netherlands (2013) [72].

In 2009, the same authors described a practical synthetic pathway to such porous TiO_2_ monoliths, envisaging chromatographic use [71]. The process also involved strongly acidic conditions, but also the use of PEO as a phase separation inducer and NMF as a proton scavenger. NMF is known for gently increasing the solution’s pH and PEO was added to counterbalance the effect of propanol release during hydrolysis, which weakened the tendency for phase separation. They addressed typical issues in monolithic TiO_2_ gels, namely poor control of the mesoporous structure and shrinkage (deformations and/or crack formation), utilizing high-temperature aging treatments of the wet gel in the mother liquor between 100 and 200 °C, as a post-gelation process to remove the solvent phase. By this approach, mesopore size was increased. Subsequent treatments to 400 °C were employed as a final step. Phosphorous-containing compounds were effectively separated using the monolithic TiO_2_ monoliths as HPLC columns. Other Ti precursors (titanyl sulfate, TiOSO_4_) and phase separation inducer agents (PVP) were employed by Li et al. in 2013 [72]. The effect of parameters, such as amount of PVP, formamide, water, and ethylene glycol (EG) were studied. This latter compound was suggested to be acting as a chelating agent. Figure 13 shows the evolution of the monoliths´ morphology for increasing PVP contents. In this case, PVP was found to be mostly present in the solvent-rich phase, which is similar to the sol-gel systems of SiO_2_ incorporated with HPAA (entropy-loss driven). This finding explains the morphologies obtained for lower amounts of PVP (Figure 13a,b), of the type of isolated macropores, because they refer to the lower volume fraction of the solvent-rich phase, besides a possible retarded phase separation phenomenon.

The anatase and rutile phases were obtained at 500 and 900 °C without disturbing the macroporosity, which enabled this compound to be potentially used in photocatalytic applications. However, a decrease of the specific surface area from 228 to 73 m^2^/g was achieved with heat treatments above 600 °C [72].

#### 5.1.2. Chelated Systems

In 2007, Backlund et al. [73] developed a new template-free method (no polymer phase separation inducer additive) where a macro and mesoporous TiO_2_ monolith was produced by using reaction rate controlling additives, namely a chelating agent (acetic acid) to control the high reactivity of the Ti precursor and a strong acid (HCl). A significant amount of water was also added to the system and the type of macroporous structure that developed was interconnected particle aggregates, maybe because gelation occurs at a later stage of phase separation. The majority of the chelating agents were removed by hydrolysis and posterior decarbonation, through gradual solvent exchange procedures.

As already mentioned, the reduction of the Ti alkoxides reactivity can be tackled either by using a strong acid, e.g., HCl or HNO_3_, and/or by adding a chelating agent, e.g., acetic acid. Additionally, other synthesis strategies were pursued to induce the homogeneous gelation of the monolithic materials.

Hasegawa et al. in 2010 developed titania homogeneous (transparent, non-macroporous) monoliths using milder conditions instead of strongly acidic conditions [74], by employing a chelating agent (ethyl acetoacetate, EtAcAc) and a mineral salt. The novelty of this method consisted of using the conjugate base of a strong acid, such as nitrate ions and halide ions, in conjunction with the chelating agent, which further stabilizes the chelated species and decreases the reactivity during hydrolysis. A variety of strong acid anions were tested in this work, and it was found that the ability of salts to suppress the too rapid sol-gel reaction strongly depended on the electronegativity of the anions and valence of the cations. NH_4_NO_3_ was concluded to be the best salt to prevent Ti atoms from being exposed to nucleophilic reactions. The template-free reaction was reported to occur in a nearly neutral condition in one-pot and the gelation could be controlled, yielding homogeneous monolithic gels without macroporosity. In order to fabricate hierarchically porous TiO_2_ monoliths, this was followed by the same authors, by incorporating in the batch containing TiPOT, 1-propanol, and EtAcAc, a phase separation inducer additive, PEO [69,70].

### 5.2. Zirconia and Alumina Macroporous Monoliths

Zirconium has a larger positive partial charge than silicon and titanium, being more predisposed to a nucleophilic attack and exhibiting greater reactivity. Besides the higher reactivity exhibited by Zr alkoxides, ZrO_2_ monoliths also exhibit lower thermal stability than SiO_2_ or TiO_2_ ones, exhibiting pore collapse when submitted to temperatures above 400 °C. In 2008, Konishi et al. were able to tackle these barriers and reported the production of thermally stable macro and mesoporous crystalline ZrO_2_ monoliths [75]. They first slowly induced the condensation of hydrolyzed zirconium propoxide (9.36 g), using NMF (2.88 g), in a strongly acidic media (2.3 g of 65 wt% aq. HNO_3_), which, as already explained, leads to a more gradual pH increase than formamide. PEO was added at contents ranging from 0.04 to 0.15 g. Secondly, to increase the monolith’s thermal stability, an alcoholic solvent exchange, followed by heat treatment, was applied to the wet ZrO_2_ gels. The authors were able to prove, for this composition, that crystallinity and mesoporosity could be modified without affecting the macroporous morphology. These findings were applied to the synthesis of porous TiO_2_ monoliths by the same team [71].

**Figure 14:**
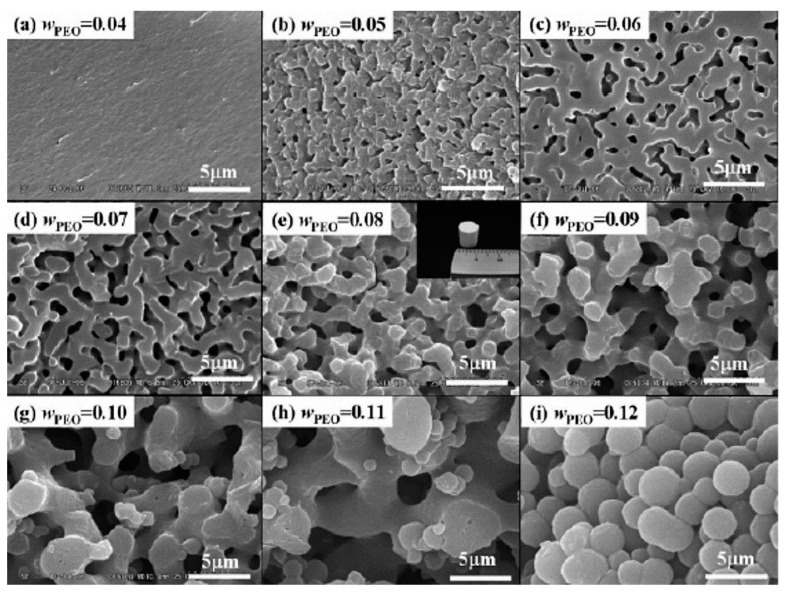
SEM photomicrographs of Al_2_O_3_ dried monoliths prepared with different amounts of phase separation inducer additive, PEO (1,000,000 Mw). Reprinted (and adapted) with permission from Tokudome Y, Fujita K, Nakanishi K, Miura K, Hirao K (2007) Synthesis of monolithic Al_2_O_3_ with well-defined macropores and mesostructured skeletons via the sol-gel process accompanied by phase separation. Chem Mater 19: 3393–3398, doi:10.1021/cm063051p [76]. Copyright 2007 American Chemical Society.

Tokudome et al. [76] (in 2007), Wu et al. [77] (in 2014), and Guo et al. [78] (in 2015) were able to demonstrate the preparation of hierarchical macro/mesoporous alumina (Al_2_O_3_), zirconia (ZrO_2_), and magnesia and yttria stabilized ZrO_2_ monoliths. They used a milder reactional medium (without the addition of strong acids or bases), an epoxide (PO)-driven approach, together with a phase separation approach (PEO) and cheaper metal sources (ionic precursors): aluminum chloride hexahydrate (AlCl_3_·6H_2_O) and zirconium chlorides, which are not as reactive as the corresponding metal alkoxides [78]. Macropore morphologies are shown in Figure 14 for alumina monoliths prepared with increasing amounts of PEO. As explained in Section 4, this synthetic approach was based on the direct condensation of the ionic precursor promoted by pH increase, after the ring-opening reaction of PO.

However, in the synthesis of 100% ZrO_2_ materials, even when using anhydrous Zr chloride, the high gelation rate of Zr^4+^ upon the ring-opening reaction still caused ZrO_2_ oligomers to quickly polymerize with formation of the 3D Zr–O–Zr gel network, inhibiting the rearrangement with PEO for phase separation. In order to prevent such a quick solid gel network formation, the polymerization rate of zirconia oligomers was slowed down by adding, to the primary precursor, magnesium or yttrium precursors, which have a low tendency to form a network by the epoxide-driven sol-gel method [77]. NMF has also been employed to promote the phase separation phenomenon [75], i.e., since it slows down the gelation process, it allows the diffusion of PEO moieties toward the oligomers, as well as the formation of gel-rich and solvent-rich phases.

The just described mechanisms, followed in [76,78], are similar to that used for achieving macroporous cordierite monoliths [60], where the best compromise between PO and PEO contents, i.e., gelation and phase separation time, was achieved to generate interconnected macropores and the co-continuous skeletons of Al_2_O_3_ or ZrO_2_. By Differential Thermal Analysis (DTA)/Thermogravimetry (TG) and Fourier Transform Infrared (FTIR) spectroscopy, it was possible to conclude that PEO was present on the dried gels. In this case, PEO is expected to be absorbed on the surface of ZrO_2_ oligomers through hydrogen bonds [78] and, therefore, increase the hydrophobic-hydrophilic repulsion with the solvent phase, causing phase separation. A solvothermal process using an ethanol solution of ammonia was found to tailor the mesopores and increase the specific surface area from 171.9 to 583.8 m^2^/g [78]; additionally, tetragonal and monoclinic ZrO_2_ crystals were precipitated at 400 and 600 °C, respectively.

In 2016 and 2017, the same authors extended this method to produce yttria-stabilized zirconia (YZA) and barium zirconate, obtaining xerogels with high porosity and surface area [79,80]. In this case, formamide was also added as a gelation agent. The amount of PEO, PO, water, or ethanol was made to vary, while the remaining quantities were kept constant: ZrOCl_2_·8H_2_O (1.610 g), formamide (0.2 mL), and EG (0.4 mL). This latter compound was found to form complexes with Ba^2+^ or Y^3+^ ions, which, together with the weak hydrogen bonds established between PEO and the inorganic oligomers, results in a distribution of PEO among the solvent-rich phase, during phase separation (entropy-driven process). This is contrary to what is observed in reference [78], where no EG is used. The sol-gel/phase separation process is schematically represented at [80]. Optimized amounts of PEO and PO were found at 0.1 g and 0.36 mL, respectively, and it should be stressed that TMOS or TEOS were employed for solvent exchange. X-ray diffraction (XRD) enabled us to conclude that the BaZrO_3_ phase was formed at 1100 °C with a few monoclinic ZrO_2_ phases, and cubic YZA was formed at 1200 °C for a high yttria doping amount.

### 5.3. Other Non-Siliceous Macroporous Monolithic Systems

**Figure 15:**
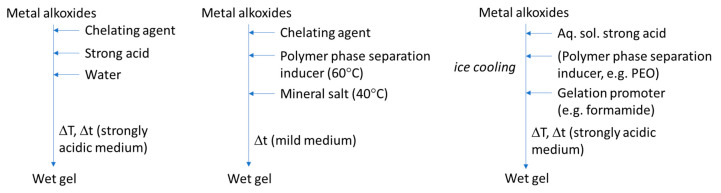
Representative synthesis schemes, where metal alkoxides are employed, showing specific variations for the achievement of macroporous monoliths of non-siliceous oxide composition, based on the sol-gel/phase separation method (Marques et al., original schemes).

The titania, zirconia, and alumina systems were the first synthesized, non-siliceous single oxides, by the sol-gel/phase separation method, and the most studied in the literature; thus, they can be considered as the baseline studies regarding single oxide monoliths. A scheme of some of the most representative synthesis strategies is shown in Figure 15, where metal alkoxides are employed.

**Figure 16:**
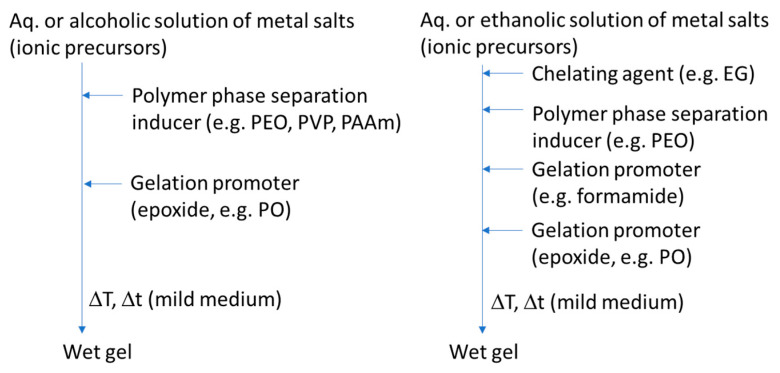
Representative synthesis schemes, showing specific variations, where metal salts (ionic precursors) are employed for the achievement of macroporous monoliths of non-siliceous single- and multi-oxide compositions, based on the epoxide-mediated sol-gel/phase separation method (Marques et al., original schemes).

When less reactive ionic precursors (metal salts) are used, synthesis strategies involve, most of the times, an epoxide-mediated method, combined with polymerization-induced spinodal decomposition, as shown in Figure 16, where two of the most representative synthesis schemes are displayed. Macroporous oxide monoliths of compositions Al_2_O_3_, TiO_2_, ZrO_2_, Cr_2_O_3_, MgO, and Fe_2_O_3_, among others, have been successfully prepared by these approaches [72,76,78,81,82,83], as well as zirconates [80,84], spinels [85,86,87,88], titanates [89], etc.

As stated before, the precursors of high-valence elements (Si, Ti, Zr, Al, etc.) are found in the form of alkoxides or inorganic salts. The latter, when in aqueous solutions, exists as hydrated ions, has a strong acidity, and can experience sol-gel reactions. However, when dealing with low-valence elements (Fe^2+^, Co^2+^, Ni^2+^, Zn^2+^, Mn^2+^, etc.), the control of hydrolysis and polycondensation is more complex; hydrolysis activity is weaker and the formation of particles to generate 3D frameworks is more difficult.

Epoxides have shown to play two roles, either as (i) acid scavengers to raise the solution pH homogeneously and promote polycondensation or induce the coordination with metal cations [90] or as (ii) phase separation inducers, via coordination to metal cations, resulting in low solubility and low compatibility oligomers in the mixed solvent [90].

An adaptation of the epoxide-mediated sol-gel process could enable the synthesis of more homogeneous porous monoliths derived from low-valence metal–salts, i.e., divalent oxidation state metals. As far as we are concerned, Gash et al. [91] (in 2004) were the first to report the synthesis with these elements, for the preparation of NiO_2_ aerogels. The difficulty of this synthesis is due to the low electronegativity of the central metal atoms and high positive partial charge of the cations, which results in the heterogeneity of the resulting monoliths. The role of the metal–salt counter ion was found to be very important. Lu et al. [92] addressed this issue and were able to obtain homogeneous gelation for three kinds of low-valence metal–salts. They studied factors such as the concentration of the polymer (Cu-system), the Mw (Co-system), and the amount of HCl (Mn-system), regarding the 3D interconnected macroporosity of the resulting monoliths. Their strategy was based on preparing brominated metal alkoxides, achieved by reaction of metal bromides (MBr_2_ with M = Cu, Co, and Mn) with epichlorohydrin in N,N-dimethylformamide (DMF), by a ring-opening reaction, whose kinetics could be controlled by ice cooling. Such brominated alkoxides (Br-M-OR), with a highly electronegative Br ligand and reduced negative charge on oxygen atoms, reduced the rates of hydrolysis and polycondensation and allowed preparation of a homogeneous composite oxide gel. PEO and PVP were employed as phase separation inducers (Cu-system) and tend to locate at the solvent-rich and gel-rich phases, respectively. For the Co-system, higher Mw PEO (up to 600,000 Da) had to be employed for phase separation to occur at an optimum level. For the Mn-system, the amount of aqueous solution in HCl was varied, keeping the amount of PEO (100,000 Da) and PVP (40,000 Da) constant. HCl promoted hydrolysis and polycondensation, resulting in the freezing of the structure at earlier stages of phase separation phenomenon.

Table 3 shows specific synthesis examples for different types of non-siliceous single oxide porous monolithic materials.

Mixed metal oxides with a spinel structure (AB_2_O_4_), such as NiAl_2_O_4_, exhibiting hierarchical pore structures [85], have also been implemented in the field of heterogeneous catalysis. The small mesopores can act as reaction pores and significantly increase the specific surface area. On the other hand, due to the presence of macropores, a very efficient mass transfer to and from the reaction pores can be achieved, avoiding the diffusion issues often present during catalytic reactions. Kido et al. [95] prepared different spinel compositions in the form of macroporous monoliths, using the epoxide method, and they found that when Fe^3+^ and Zn^2+^ inorganic salt precursors were used to prepare those monoliths, the obtained structure gradually transited from a cocontinuous macroporous structure to the accumulation of particles with the increase of the binary precursor content. This was due to the large difference in the hydrolysis rate between Fe^3+^ and Zn^2+^ (hydrolysis rate constants pK_a_(Fe^3+^)(2.2) and pK_a_(Zn^2+^)(9.2)). When such a difference is in place, the element with a faster hydrolysis rate tends to form in advance of the corresponding products, which leads to a large difference in local structure and composition.

**Figure 17:**
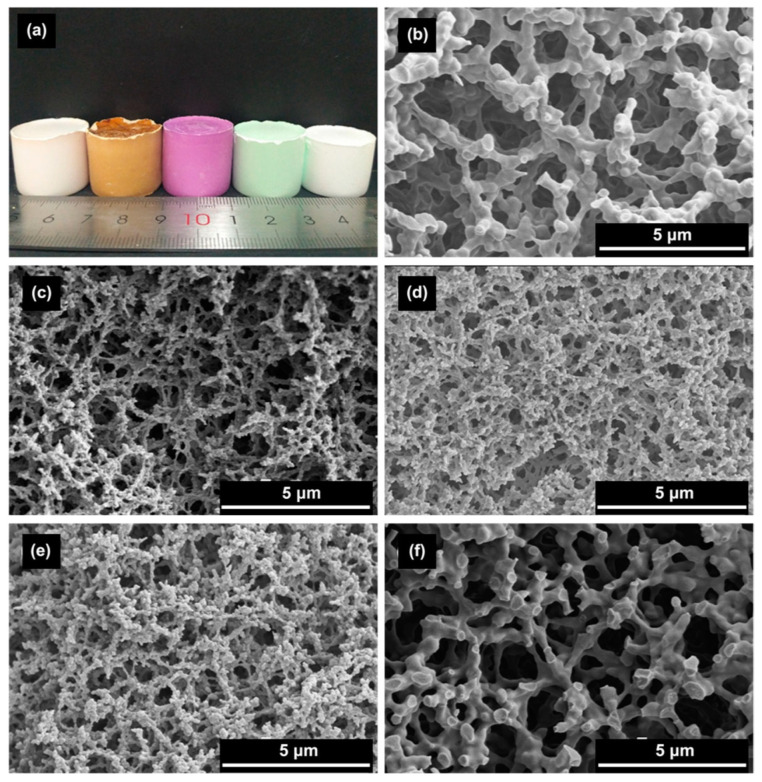
(**a**) Appearance of typical xerogel transition metal hydroxide monoliths. Corresponding SEM photomicrographs exhibiting the monoliths´ microstructure, derived from precursors of following low-valence elements: (**b**) Mn^2+^, (**c**) Fe^2+^, (**d**) Co^2+^, (**e**) Ni^2+^, and (**f**) Zn^2+^, respectively. Reprinted from the open access paper by F. Liu, D. Feng, H. Yang, X. Guo, “Preparation of macroporous transition metal hydroxide monoliths via a sol-gel process accompanied by phase separation.”, Sci. Rep. 10 (2020) [96]. http://creativecommons.org/licenses/by/4.0/ (accessed on 15 March 2021).

Transition metal hydroxide porous monoliths have also been developed recently (Table 4), in the presence of PO and HPAA [96], involving low-valence elements (Mn^2+^, Fe^2+^, Co^2+^, Ni^2+^, and Zn^2+^). Inorganic metal–salts were employed in this case, which undergo hydrolysis to form hydrated metal ions complexes with water molecules. These will further polycondense to form particles, which will combine with the HPAA macromolecules forming porous gel frameworks by phase separation (entropy-driven system). Figure 17 displays the pore morphology obtained for varied transition metal hydroxide monoliths synthesized by the epoxide method. The effect of varied synthesis parameters was studied in this work [96]. Larger amounts of water promote the hydrolysis reaction of the Zn precursor and, therefore, an increased number of OH groups at the surface of the oligomers, establishing strong H bonds with HPAA, delaying phase separation, and resulting in finer phase-separated domains. On the other hand, larger amounts of glycerol inhibit hydrolysis reactions, leading to an earlier phase separation and coarser skeletons. Additionally, larger amounts of precursor lead to coarser skeletons and broader macropore size distribution. Binary compositions involving these elements were studied and, interestingly, it was found that the combination of elements with significant difference in hydrolysis rate constants failed to give a cocontinuous, macroporous structure. An example for such case is the Zn^2+^ and Mn^2+^ systems, where the difference in the hydrolysis rate between Zn^2+^ and Mn^2+^ is 1.6. While for Ni^2+^ and Co^2+^ and Zn^2+^ and Fe^2+^ system, of low difference in hydrolysis rate (0.9 and 0.7), binary macroporous transition metal composites were possible to obtained via the sol-gel process accompanied by phase separation.

Table 4 shows specific synthesis examples for different types of non-siliceous materials (oxides and non-oxides), including recent developments on carbon (C), C/oxides, and metal organic frameworks (MOFs).

C and C/oxide-based monoliths, in particular C/TiO_2_ nanocomposites, are presently under investigation for applications, such as catalysis and photocatalysis, energy storage, dye-sensitized solar cells, anode materials for lithium-ion batteries, etc. Porous TiO_2_ monoliths can be synthesized and/or infiltrated with a C source, followed by carbonization, or TiO_2_ can be in situ generated from TiCl_4_ or Ti(OnBu)_4_ sources, within a phenolic resin. For instance, hierarchically porous TiO_2_/C composites, with in situ distributed C, were synthesized by using PVP as a phase separation inducer and C source [102], after calcination of the dried gels in an argon atmosphere at 500–800 °C. Formamide was used to promote gelation. This material was shown to have good electrochemical performance with fast Li-ion diffusion and high electronic transport efficiency, but specific surface area was found to decrease from 170 to 7 m^2^/g, when calcination temperature was increased from 500 to 800 °C. Resorcinol and formaldehyde have also been used as organic C sources, however, the achievement of ordered mesopores or specific hierarchical structures was not straightforward.

Porous monoliths of C-based compositions and metal organic frameworks (MOFs) have not been prepared following the exact approaches represented in Figure 15 or Figure 16; instead, they have derived from self-assembly-induced phase separation processes, as described below.

Hasegawa et al. (2016 and 2020) [27,103] prepared RF monoliths and C monoliths (by subsequent carbonization) by controlling three phenomena, which are made to occur in parallel: polymerization, self-assembly, and phase separation by spinodal decomposition. In this process, there is polycondensation of resorcinol and formaldehyde and cooperative self-assembly of RF oligomers; F127 takes place concomitantly with the occurrence of spinodal decomposition, resulting in a macroporous skeleton with embedded mesopores. This is a kind of self-assembly-induced phase separation process. Basically, the hydroxyl groups on RF oligomers interact with the hydrophilic moieties of F127 via hydrogen bonding, leaving their hydrophobic region facing outward. It should be noted that TMB and BzOH are also employed to enlarge (swell) the RF/F127 micelles and as a cosurfactant, respectively, and triethylene glycol (TEG) is used as a solvent with high compatibility with the micelles to not limit the extent of phase separation. The relatively high hydrophobicity of the RF/F127 units promotes macroscopic phase separation in aqueous media. The growth of the phase-separated RF/F127 domains stops at the moment of the sol-gel transition. The porous RF monoliths are then obtained, which are readily converted to the C monoliths, preserving the bimodal pore system. KCl was also used, in some cases, [27] to improve the mesopore arrangement; however, it was still not enough to achieve an ordered mesostructure with a periodicity at long-range.

**Figure 18:**
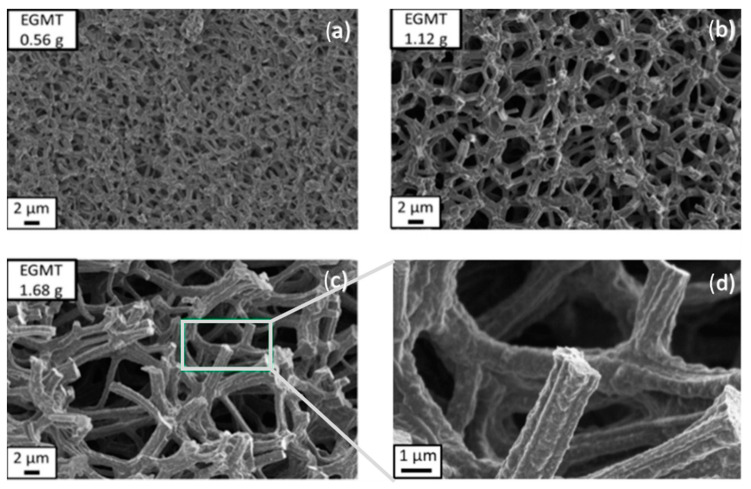
SEM photomicrographs of C/TiO_2_ with increasing amounts of TiO_2_ (Ti precursor EGMT): (**a**) 0.56 g; (**b**) 1.12 g; (**c**) 1.68 g; (**d**) magnification of (**c**). Reprinted with permission from J. Schoiber, C. Koczwara, S. Rumswinkel, L. Whitmore, C. Prehal, F. Putz, M. S. Elsaesser, O. Paris, N. Hüsing, Chempluschem, Copyright © 2021 WILEY-VCH Verlag GmbH & Co. KGaA, Weinheim [104].

Schoiber et al. [104], from Hüsing group, developed (in 2021) a methodology also based on self-assembly-induced phase separation, involving (ethylene glycol)-modified titanate (EGMT) as Ti precursor to achieve hierarchical porous RF/TiO_2_ materials, which are converted into C/TiO_2_ composites via carbonization. The morphology of the monoliths synthesized with increasing amounts of the Ti precursor is shown in Figure 18.

A comparison, with the method proposed by Hasegawa et al. [27], was discussed in this work. The advantage reported for EGMT is its stability, i.e., the reactivity towards water was not too high and there was good solubility in the RF system, and also the release of EG, compatible with F127, instead of monohydric alcohols during hydrolysis. The mechanism proposed is: (a) formation of hexagonal ordered F127 micelles at room temperature (RT), (b) polycondensation of resorcinol and formaldehyde around F127 micelles at 60 °C, (c) further polycondensation of resorcinol and formaldehyde and sol-gel reaction of Ti precursor at 60 °C, and (d) carbonization of RF/TiO_2_ composite to obtain C/TiO_2_ at 850 °C. It was shown in this work that the lower the polarity of the solvent employed, the better the quality of the hierarchical structure obtained. TEG exhibited better results than diethylene glycol (DEG) or EG. Since protons (pH value) play a role in the catalysis of the condensation reactions, but also in the interaction between the solvent molecules, their concentration must be carefully adjusted to the solvent used. The hierarchically organized porous C/TiO_2_ monolithic materials were obtained using a good combination of diol-based solvent and pH value, as well as the control between phase separation induced by an oligomeric block copolymer (F127) and gelation of RF.

The sol-gel/self-assembly-induced phase separation process was also applied to develop porous monolithic MOFs gels, with trimodal porosity (at the micro, meso, and macroscale). The focus was on a Zr-based UiO-66 type MOF with the formula [Zr_6_O_4_(OH)_4_(1,4-benzenedicarboxylate)_6_], reported by Hara et al. [105]. It started by the self-assembly of the Zr moieties (from an inorganic precursor, namely ZrOCl_2_·8H_2_O) and terephtalic acid, namely 2-aminoterephthalic acid (BDC-NH_2_). The latter is used as organic linker to obtain the Zr-terephtalate-based MOF (Zr-BDC-NH_2_ clusters, hydrophilic) with good solubility in DMF, to which self-assembly-induced phase separation was applied. A phase separation inducer (polypropylene glycol, PPG), hydrophobic, was added, and a reorganization process of such clusters at the microscale, induced by acetic acid, was able to convert the macroporous structure from low-crystalline framework to crystalline UiO-66 (macroporous UiO-66-NH_2_). PPG was found to not strongly interact with Zr-BDC-NH_2_ and it was assumed to be distributed both at the solvent, and gel-rich phases, acting as co-solvent [105].

## 6. Macroporous Microspheres

**Figure 19:**
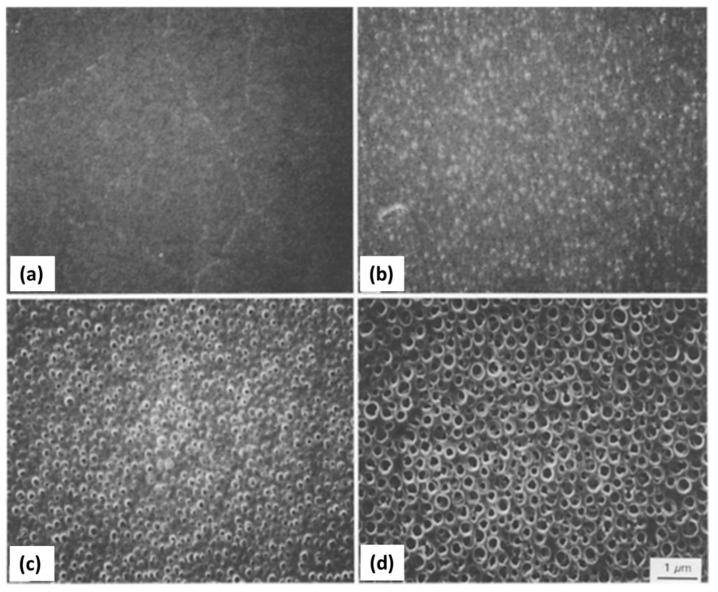
Morphology of TiO_2_ thin films prepared with increasing amounts of phase separation inducer, PEO (1800–2200 Mw), and obtained by dip-coating at 100 mm/min withdrawal speed: (**a**) 0 g; (**b**) 0.5 g; (**c**) 1 g; (**d**) 2 g PEO. Reprinted by permission from Springer Nature Customer Service Centre GmbH: Springer, J. Mat. Sci., Morphology of thin anatase coatings prepared from alkoxide solutions containing organic polymer, affecting the photocatalytic decomposition of aqueous acetic acid, Kato K, Tsuzuki A, Torii Y, Taoda H, Kato T, Butsugan Y, ©1995 Chapman & Hall [106].

The previous chapters give an extensive view on the different synthesis strategies, based on sol-gel/phase separation methodology, targeting macroporous or hierarchical macro and mesoporous monolithic products. Thin films produced by the same approach, and exhibiting similar kinds of porosity, are not so frequent in the literature, but can also be found (for example, TiO_2_ (Figure 19) and SrTiO_3_ thin films [106,107,108], where PEO was used as a phase separation inducer additive).

However, monoliths and thin films may pose some limitations in specific applications, due to their fixed shape. Controlling porosity through phase separation and transferring this know-how into the realm of microscopic spheres is of great importance, but brings specific challenges, since a further phenomenon, i.e., spheroidization by soft templating methods, needs to be controlled in the synthesis process. For instance, emulsion flocculation, sedimentation, droplet coalescence, etc., are all phenomena that can have a strong impact on the macropores formation. Moreover, all the complex processes described previously, such as phase separation thermodynamics and sol-gel kinetics, must occur within the tiny droplets of an emulsion, which may pose geometrical or space limitations for generating the desired interconnected macroporosity. Yanagisawa et al. studied the spontaneous patterning of polymer microgels by confining a polymer blend (PEG and gelatin) within microspheres, showing the interplay among phase separation, wetting and gelation in the presence of droplet confinement [109].

As for silica and non-siliceous-based porous microspheres, these are promising materials, since they can act as support materials and are of great interest in many fields, ranging from chromatography to catalysis, including storage and delivery, phosphor materials (white-light emitting diodes), antibacterial activity, and enzyme fixation [110]. One further example is the potential use of silica-based porous microspheres as support materials for chemical immobilization to replace polymeric microcapsules, which are in the scrutiny since they are regarded as microplastics. Many industries are looking for replacement solutions, and this type of porous microsphere might be a potential candidate.

The published works on interconnected cocontinuous macroporous microspheres, possible to obtain by the sol-gel/phase separation method, are very scarce. The development of microspheres with varied pore morphology has been achieved mainly by the sol-gel route, combined with self-assembly technology [111], emulsion process [2,112], dispersion process [113], nanocasting [114,115], or using hard templates, such as polystyrene (PS) beads to reach the desired macroporosity [116,117].

Regarding template-free synthesis protocols, mesoporous microspheres of composition InVO_4_ were developed through Oswald ripening and self-assembly aggregation of preformed InVO_4_ NPs, in the presence of CTAB, which binds the NPs together through electrostatic interaction after their aggregation has taken place. These porous microspheres were reported to exhibit enhanced visible-light absorption ability [118], however poor cohesiveness (not directly assessed in this work) might be an issue for these materials. In situ growth of TiO_2_ NPs on these microspheres was performed to achieve TiO_2_/InVO_4_ nanocomposite microspheres [118], with high photocatalytic activity and recycling ability. Also targeting photocatalysis, the same authors have studied composites made from the mixture of the porous InVO_4_ microspheres with C quantum dots under reflux [119].

Hollow (core-shell) microspheres of different compositions, loaded with nanoparticles (NPs) within their mesoporous-structured shell have been fabricated for applications, such as light diffusers and magnetic-assisted separation agents. One example is the synthesis of AgNPs@silica hollow microspheres (microcapsules), which started from PS beads, as a template, and made use of polyvinylpyrrolidone (PVP) as an Ag^+^ reduction agent and mesoporosity inducer at the shell. Both PVP and PS were removed by calcination at the end of the synthesis [111]. The resulting AgNPs@silica core-shell microspheres were able to protect the immobilized AgNPs located within the microspheres´ shell and, simultaneously, maintain antibacterial activity for long periods, through the release of the remaining Ag^+^ ions in aqueous solution.

Nanocrystalline Fe_2_O_3_, SnO_2_, ZrO_2_, and Mn_2_O_3_ microspheres with additional internal mesopores and/or macropores have been prepared by nanocasting using commercially available mesoporous silica spheres of different diameters, as the template. These were impregnated by the metal–salt solutions and silica was leached out in HF or NaOH solution [114]. Other works also involved hard-templating methods for the preparation of metal or metal oxide porous microspheres, such as pre-formed microspheres made of poly(GMAco-EGDMA), a polymer of glycidyl methacrylate (GMA) cross-linked with ethylene glycol dimethacrylate (EGDMA), which were functionalized with ethylenediamine (EDA) and infiltrated with Si, Ni or Zr precursors to give porous metal oxide, or metal microspheres, after calcination [120,121,122], to be applied as catalysts, or in chromatography columns. It should be stressed that these techniques, despite resulting in spheres of good quality and uniform size distribution, involve several synthesis steps and expensive reagents and are time consuming (more than 2 days of synthesis).

A different strategy was introduced by Mori et al., in 1993 [123], where a water-in-oil (W/O) emulsion was prepared to obtain microspheres of SiO_2_-B_2_O_3_-Na_2_O composition, with some degree of macroporosity, by extracting, after a heat treatment at 600–750 °C for 16 h, soluble parts of the glass using sulfuric acid. In this process the W phase contained TMOS and B and Na precursors, together with DMF and a high amount of water (H_2_O/TMOS = 20), while the O phase was made of kerosine and Span 80. The heat treatment of the resulting spheres promoted phase separation, which together with a subsequent extraction of the soluble phase (mainly based on boron and sodium) by sulfuric acid, led to the generation of interconnected macroporosity, especially at the surface of the spheres [123]. In fact, phase separation was found to proceed more effectively, when temperature was raised up. This technique does not involve the type of phase separation by spinodal decomposition (chemical cooling) reported in Section 2, Section 3 and Section 4 of the present paper.

### 6.1. Macroporous Microspheres by Sol-Gel/Phase Separation

Different types of emulsions and of sol-gel/phase separation phenomena are among the strategies that lead to macroporous microspheres, either with incontinuous inner macroporosity (multicavities), or with interconnected continuous macroporosity, or even with hierarchical interconnected porosity at different length scales.

#### 6.1.1. Multicavities/Incontinuous Inner Macroporosity

**Figure 20:**
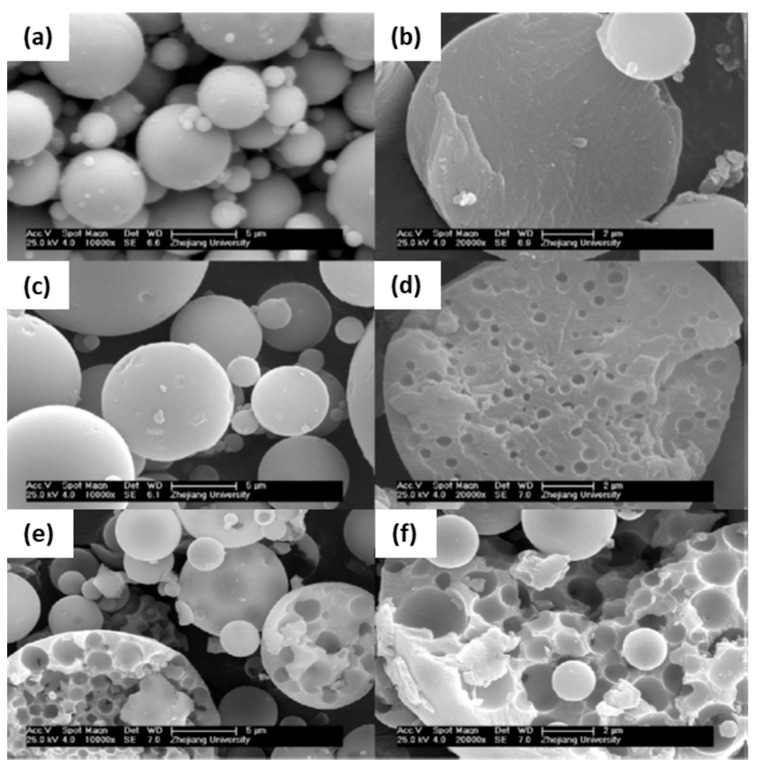
SEM photomicrographs of TiO_2_ microspheres exhibiting inner incontinuous macroporosity, prepared by O/W emulsion method combined with sol-gel transition and phase separation. The larger the amount of phase separation inducer (PVP), the larger the pore volume and size. (**a**,**b**) 0 g PVP; (**c**,**d**) 0.190 g PVP; (**e**,**f**) 1.903 g PVP. Reprinted from Chinese Chem. Lett., Vol. 25, W. W. Cai, H. Yang, X. Z. Guo, A facile one-step route to synthesize titania hollow microspheres with incontinuous multicavities, Pages 441–446, Copyright (2014), with permission from Elsevier [125].

Oil-in-water (O/W) emulsions combined with sol-gel processing in the presence of PVP additive have been employed to create metallic oxide microspheres, with a smooth surface, exhibiting multicavities in their core. The oil phase is composed by 1-octanol, metal alkoxide and PVP. The latter one was found in this case to induce a nucleation growth phase separation phenomenon, followed by sol-gel transition and drying, which resulted in the incontinuous inner macroporosity. Potential applications for these microspheres are on heat insulation and separation (liquid chromatography) [124]. Cai et al. (2014) [125] reported on this synthesis for the preparation TiO_2_, ZrO_2_ and Al_2_O_3_ microspheres [124,125,126] with incontinous multicavities of sizes (diameter) in the range of 150 nm to 1.3 µm. The larger the PVP amount, the larger the cavities´ size. Their morphology (inner macroporosity) is shown in Figure 20. for increasing amounts of PVP.

**Figure 21:**
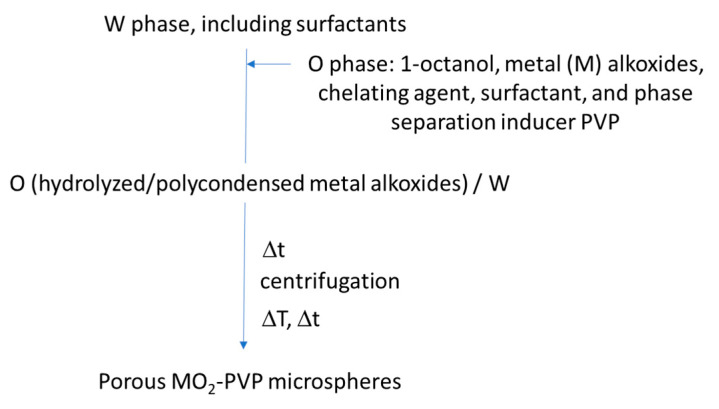
Representative synthesis flowchart, employing O/W emulsification as soft template, and sol-gel/phase separation, for the achievement of metal oxide microspheres with incontinuous inner macroporosity [125] (Marques et al., original schemes).

**Figure 22:**
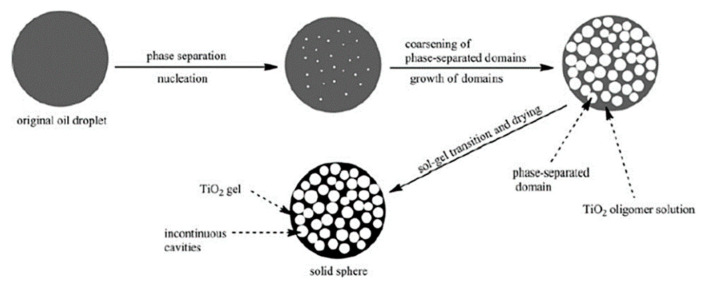
Representation of the mechanism for the formation of TiO_2_ microspheres with incontinuous inner macroporosity. Reprinted from Chinese Chem. Lett., Vol. 25, W. W. Cai, H. Yang, X. Z. Guo, A facile one-step route to synthesize titania hollow microspheres with incontinuous multicavities, Pages 441–446, Copyright (2014), with permission from Elsevier [125].

As shown in Figure 21 the oil (O) phase is composed by 1-octanol, metal alkoxide and PVP, as well as a chelating agent, to suppress the fast hydrolysis of the metal alkoxides, and a surfactant (Span 80), used to stabilize the oil droplets of the emulsion and to preserve their spherical shape [125]. A polar solvent was selected (1-octanol) to facilitate the diffusion of water in the O phase and accelerate the sol-gel reactions. Surfactants alkylphenol ethoxylates (OP-10) and sodium dodecyl sulfate (SDS) were added to the water (W) phase of the emulsion to lead to a more homogeneous particle size distribution. Similar to the works reporting on porous monoliths, the phase separation was induced by the strong interaction between PVP and the metal oxide oligomers, which leads to a hydrophilic–hydrophobic repulsion between the oligomers and the solvent. In fact, the hydroxyl groups of Span 80 are known to establish H-bonds with the amide groups of PVP, so that Span 80 locates at the interface of the phase-separated domains. The influence of the heat-treatment on the crystalline phase and the porous structure was also investigated in these works. A simplified scheme of the synthesis is shown in Figure 22.

#### 6.1.2. Interconnected Macroporosity

Yang et al. (2006) [127] made some of the first attempts to achieve silica microspheres with mesoporosity, which also exhibited some macroporosity, but still with some space for improvement, especially in terms of spherical morphology and pore size and shape control. They employed PEO and F127 to induce porosity formation in a pre-hydrolyzed silica sol, and a mechanism based on a pH-induced rapid colloid aggregation, by addition of triethylamine (TEA) or trioctylamine (TOA), for the formation of the silica spheres, since the positive charges on the surface of the silica primary particles were counteracted by the alkali. All of this inside the water phase droplets of a water-in-oil (W/O) emulsion. The addition of PEO accelerated gelation of the sol and the formation of silica spheres, mainly through reducing the primary particle size. F127 acted as surfactant to produce mesopores, while PEO acted as auxiliary template to produce macropores.

**Figure 23:**
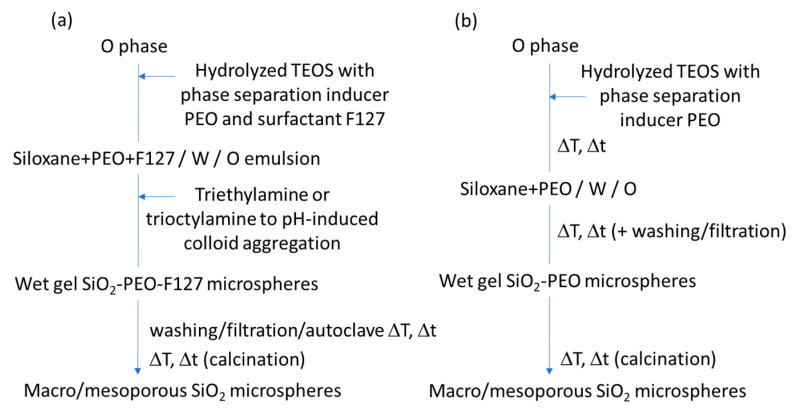
Representative synthesis flowcharts, employing W/O emulsification as soft template, and sol-gel/phase separation, for the achievement of macro/mesoporous SiO_2_ microspheres: (**a**) method developed by Yang et al. [127]; (**b**) method developed by Shi et al. [112]. The O phase is composed by paraffin oil and Span 80. (Marques et al., original schemes).

Silica microspheres with hierarchical interconnected macro/mesoporosity were obtained by Shi et al. [112], in 2008, using the synergetic interaction of phase separation and sol-gel transition in a confined W/O emulsion droplet. These synthesis strategies are schematically represented in Figure 23.

**Figure 24:**
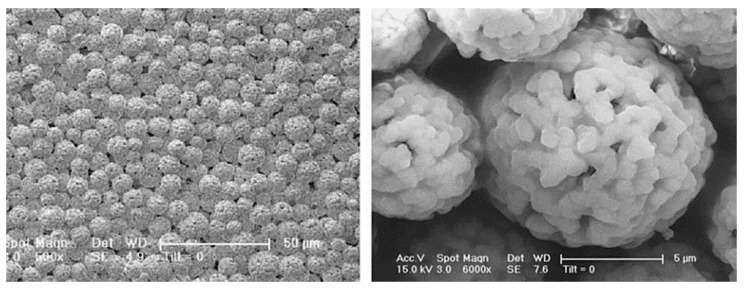
SEM photomicrographs for the SiO_2_ microspheres prepared by the W/O emulsion method combined with sol-gel transition and phase separation, using TEOS as Si precursor and PEO as phase separation inducer additive. Their narrow size distribution is due to liquid elutriation techniques, which allowed to select microspheres of diameter ranging from 6 to 10 µm. Scale bar = 50 μm (left); 5 μm (right). Reprinted from Microporous Mesoporous Mater., Vol. 116, Z. G. Shi, Y.Q. Feng, Synthesis and characterization of hierarchically porous silica microspheres with penetrable macropores and tunable mesopores, Pages 701–704, Copyright (2008), with permission from Elsevier [112].

TEOS was hydrolyzed in HCl to form silica sol (W phase) and ethanol as byproduct, to which PEO was added. Since ethanol was miscible with the O phase (paraffin oil), it was first eliminated using vacuum distillation. Emulsification was carried out by magnetic stirring, while heating at 60 °C during 20 h was employed to promote gelation and phase separation within the droplets of the emulsion. The presence of PEO was responsible for phase separation by spinodal decomposition to occur. The two transient continuous phase domains of silica-rich and solvent-rich phase coarsened as the sol-gel transition proceeded. After liquid elutriation, spheres with diameters ranging from 6 to 10 µm were gathered and proceeded for characterization. By calcination, the solvent-rich phase and PEO were burned off forming the macropores [112], which can be observed in Figure 24.

PEO was found to have two functions in the microspheres formation process: phase separation induction and porogenic function [112]. However, the small mesopores developed within the spheres´ skeleton had to be enlarged and this was achieved by a treatment with aqueous solution of NH_4_OH 0.01 M at 120 °C (dissolution-reprecipitation process). Consequently, the surface area decreased by 33%, but the pore volume increased by 50%. The mechanical strength of these microspheres is expected to be larger than that of the microspheres obtained by self-assembly, or those with incontinuous pores, in particular multicavities of large diameter and thin walls.

Over the years, similar microspheres were obtained through the described emulsion method (Figure 23b), with slight variations, such as addition of Span 80 and/or NH_4_OH to the O phase of the emulsion, and the resulting porous microspheres were applied mainly to chromatography [110,112,128,129,130,131,132,133], but also in drug delivery [134,135], adsorption [136] and composites production [137]. In this latter one, the porous SiO2 microspheres were incorporated in phenolphthalein-based poly(arylene ether sulfone) resin. Another adaptation of the technique of Figure 23, targeting hybrid SiO2 microspheres, consisted of the employment of silanes TEOS and BTME, and a slightly different O phase made of petroleum ether, Triton X-100 and Span 80 [110].

Another attempt for porous microspheres involved the previous preparation of polyethoxysiloxane, from TEOS, containing oligomeric PEG additive of 400 Mw and using ciclohexane as stabilizer, which was mixed within an acidic solution of water and ethanol [138]. The resulting microspheres were small, ca. 4–5 µm, and without macroporosity, only mesoporosity. PEG was responsible for the presence of mesopores (~5–7 nm) and a specific surface area of ca. 230 m^2^ g^−1^. Too much PEG was found to degrade the spherical shape of the particles. These microspheres were used in liquid chromatography for chiral separation of flurbiprofen axetil stereoisomers. Mesoporous microspheres of rare earth (RE) doped yttrium aluminum garnet Y_3_Al_5_O_12_ (YAG:RE^3+^), namely YAG:Ce^3+^ [139], were obtained through a fast epoxide-driven sol-gel route using inorganic metal–salts as precursor, in particular YCl_3_·6H_2_O, AlCl_3_·6H_2_O and cerium chloride heptahydrate. Formamide and PO were used, as well as F127. A heat treatment under a flowing gas mixture (5% H_2_ + 95% N_2_) for 6 h at 1000–1600 °C was carried out. The authors reported that gelation in this case was faster than in traditional epoxide sol-gel process, because of the larger amount of metal ion and PO/metal ion molar ratio. The lack of macroporosity of the resulting microspheres and their small size (ca. 2 µm) might be due to the almost immediate gelation process which occurred sooner than the onset of phase separation inhibiting it from occurring.

**Figure 25:**
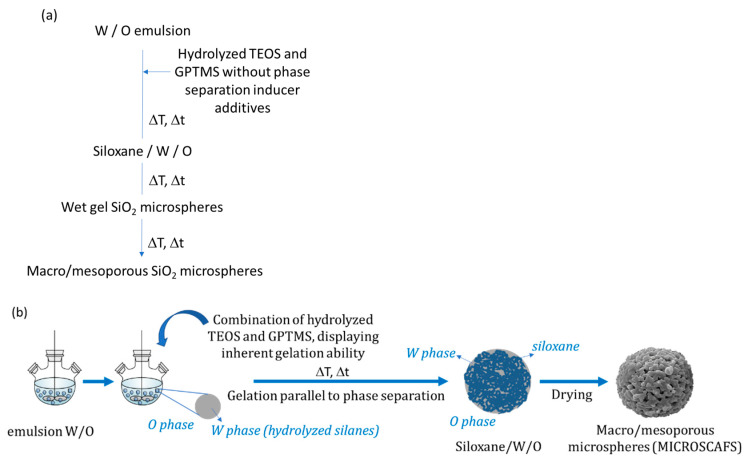
(**a**) Preparation schemes for the sol-gel derived macro/mesoporous SiO_2_ microspheres (MICROSCAFS), synthesized from TEOS and GPTMS, obtained by W/O emulsion combined with sol-gel transition and phase separation, in the absence of any gelation or phase separation inducer additive: (**a**) representative synthesis flowchart; (**b**) drawing scheme. The O phase is composed by decahydronaphthalene (decalin) and Span 80. (Marques et al., original schemes).

**Figure 26:**
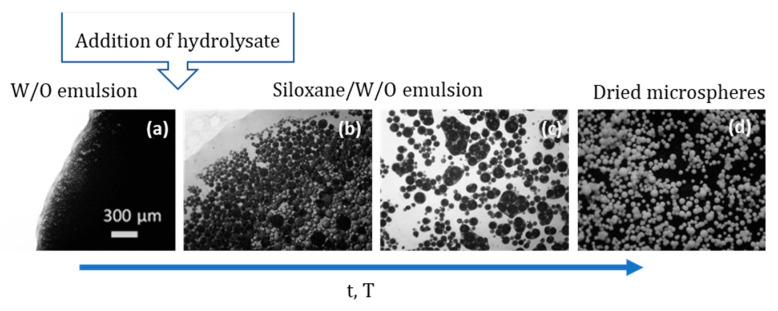
Optical microscopy photographs of reaction medium aliquots taken at different stages of the MICROSCAFS’ synthesis. (**a**) emulsion, (**b**) emulsion after addition of the hydrolysate, 1h at 65 °C, (**c**) after 1h at 80 °C, (**d**) epoxy functionalized silica-based porous microspheres dried at 40 °C. Bar scale = 300 μm (**a**–**d**). Photos acquired in transmission mode (**a**–**c**). Photo acquired in reflection mode (**d**) (A.C. Marques et al., unpublished results).

Although a few authors successfully made hierarchical macro/mesoporous spheres by using PEO, or PVP as phase separation inducer additives, and surfactants in the water phase droplets of the emulsion (Table 5), an original and simple approach was developed by Marques et al., based, as well, on sol-gel transition and phase separation between siloxane-rich domains and water-rich domains, within the droplets of a W/O emulsion, but where no specific gelation, nor phase separation promoting additives are needed. Instead, the silane combination employed (TEOS and (3-glycidyloxypropyl) trimethoxysilane, GPTMS) was selected to provide an inherent gelation capability, through the oxirane group of the epoxy silane (GPTMS) used. This method has a few similarities both with phase separation in alkylene-bridged polysilsesquixanes sol-gel systems [37], and with the epoxy-mediated synthesis approaches, before reported for monolithic macroporous gels [55], but no specific gelation additives, such as PO or formamide, are needed, nor hydrophilic polymers as templates for phase separation, which eliminates the need for calcination to get rid of the polymeric additives, and achieve the macroporous material. A simple drying of the spheres is enough to eliminate the solvent remaining within the 3D interconnected co-continuous porous structure. A simplified flowchart and scheme regarding the porous microspheres preparation is shown in Figure 25a,b, together with optical microscopy images (Figure 26) showing the evolution of the reaction medium during the synthesis, until a free-flowing powder is obtained after drying at 40 °C. A heat treatment at temperatures above 500 °C can be done just in case purely inorganic porous microspheres are desired. In the present methodology, phase separation tends to occur via siloxane formation by polycondensation of the silanol groups, but also from the epoxy ring opening reaction, being both an entropy and enthalpy-driven process.

**Figure 27:**
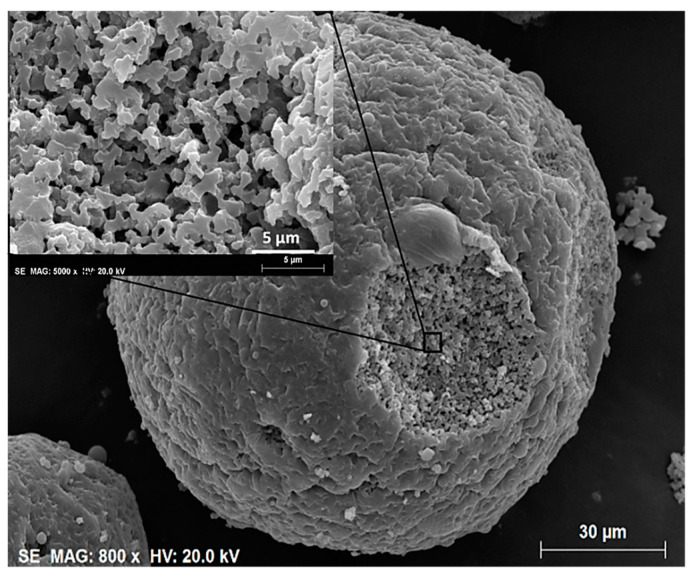
Macro/mesoporous epoxy-functionalized silica microspheres (MICROSCAFS), synthesized from TEOS and GPTMS, obtained by emulsion combined with sol-gel transition and phase separation, in the absence of any gelation or phase separation inducer additive. Reprinted from Microporous Mesoporous Mater., Vol. 261, M.V. Loureiro, M. Vale, A. De Schrijver, J.C. Bordado, E. Silva, A.C. Marques, Hybrid custom-tailored sol-gel derived microscaffold for biocides immobilization, Pages 252–258, Copyright (2018), with permission from Elsevier [140].

The first report on such synthesis occurred in 2018 [140], where the as-prepared porous epoxy-functionalized silica microspheres, shown in Figure 27 were grafted with a biocide (Econea^®^), by covalent bonding between the NH groups of the biocide and the oxirane group of the microspheres, targeting anti-fouling capability by contact.

**Figure 28:**
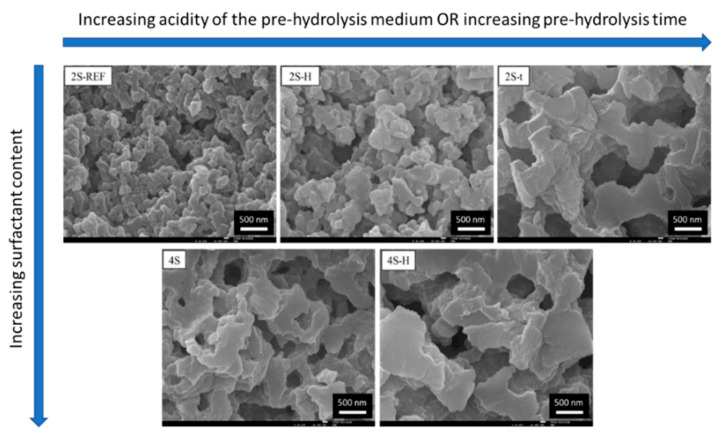
SEM photomicrographs of the microspheres’ internal structure (×30,000, bar scale = 500 nm), when varying the pH or time of the pre-hydrolysis, or the amount of surfactant, Span 80, present in the O phase of the emulsion. Reprinted by permission from Springer: Springer Nature, Journal of Sol-Gel Science and Technology, Silica-based microspheres with interconnected macroporosity by phase separation, Mário Vale, M. V. Loureiro, M. J. Ferreira, A. C. Marques [2] Copyright Springer Nature 2020.

Vale et al. [2], in 2020, studied the effect of different reactional parameters on the microspheres’ porosity for a deeper understanding of the spheres´ porosity control by this phase separation-based technique. The obtained microspheres displayed a diameter and a characteristic size ranging from 26 to 130 μm and 149 ± 11 to 485 ± 38 nm, respectively, as well as a large amount of interconnected macropores, peaked at 164–405 nm, depending on the sample, i.e., on the hydrolysis and emulsification parameters. Longer hydrolysis time, lower hydrolysis pH (increased HCl/silanes molar ratio), and higher surfactant (Span 80) content, present in the O phase, were found to lead to smaller microspheres, but more coarsened domains (increased characteristic sizes). This work suggested that the more viscous silane solution (hydrolysate), the more evolved phase separation, i.e., the more coarsened the siloxane domains are (Figure 28).

Nuclear magnetic resonance (NMR) analysis showed that most of the oxirane rings of GPTMS had opened during the microsphere’s synthesis, acting as an inherent gelation and phase separation promoting agent. However, the FTIR-ATR spectra of the microspheres dried at 150 °C revealed their hybrid character and the presence of remaining oxirane rings (not reacted). This enables the grafting of selected chemical species for any desired application. A heat-treatment of the microspheres at 700 °C was also carried out to obtain silica, fully inorganic material, composed by T and Q Si units, detected by NMR [2].

**Figure 29:**
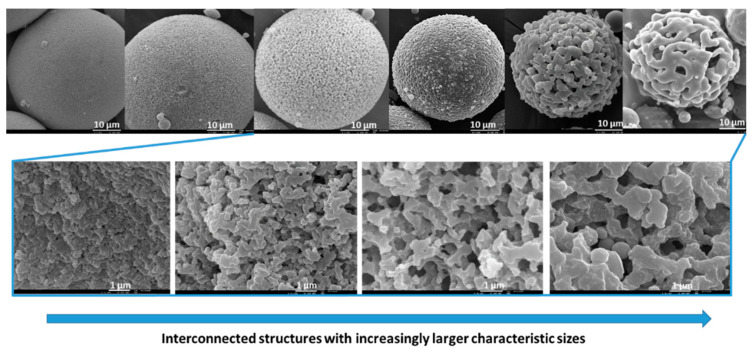
Varied morphologies obtained for MICROSCAFS, synthesized from TEOS and GPTMS, through emulsion combined with sol-gel transition and phase separation, in the absence of any gelation or phase separation inducer additive (A.C. Marques et al., unpublished results).

Microspheres with a wide variety of porous morphologies (Figure 29) were synthesized by the technique reported in [2,140]. Those differences derive from the temporal space between the “freezing” of the structure and the onset of phase separation. The co-existence of several phenomena, such as methanol and ethanol release from hydrolysis and the abundant water content inside the emulsion W phase droplets, which play opposite roles in terms of phase separation tendency and contribute to the complexity of the process. However, a fine tuning of the silanes hydrolysis parameters and emulsification conditions, including surfactants content and W/O volume ratio, allows for the achievement of a great diversity of microspheres´ morphology.

**Figure 30:**
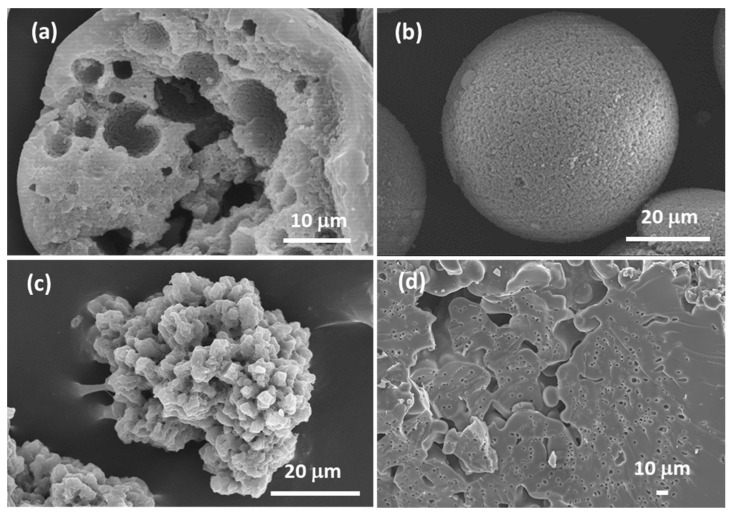
SEM images of microspheres with varied pore morphology, resulting from different freezing and phase separation onsets. (**a**) too early freezing, or retarded phase separation; (**b**) simultaneous freezing and phase separation by spinodal decomposition; (**c**) freezing at the late stage of phase separation; (**d**) macroscopic two phase structure (isolated macropores). (A.C. Marques et al., unpublished results).

**Figure 31:**
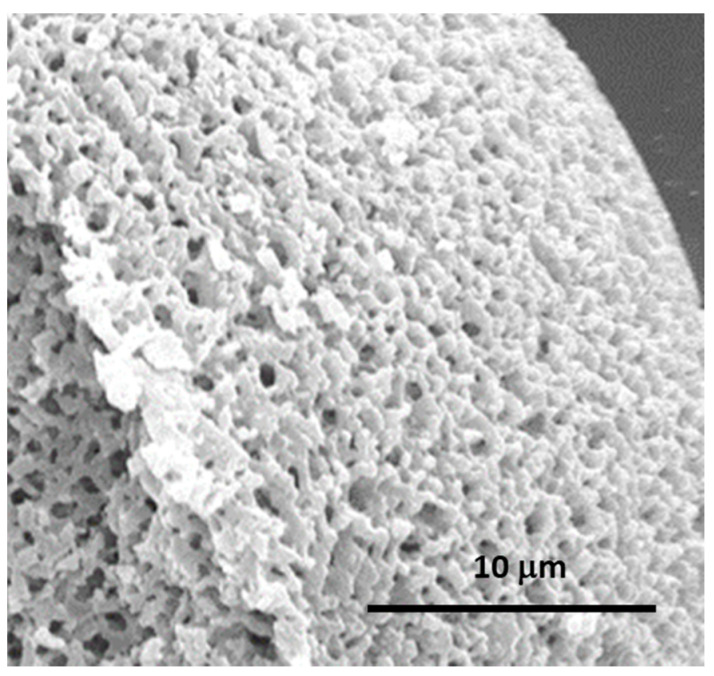
SEM image of a porous microsphere (MICROSCAF) obtained when the onset of gelation is coincident with the onset of phase separation (by spinodal decomposition). (A.C. Marques et al., unpublished results).

Figure 29 and Figure 30 exhibit microspheres with pore structures achieved at different coarsening levels. The microspheres were attained at ca. the critical composition, φc, so that phase separation by spinodal decomposition occurred, but with different freezing, or phase separation onsets. Too early freezing, or retarded phase separation, results in finer structures and holes in the structure (Figure 30a), because the growing of the continuous gel network is still in the beginning. The spheric large pores of Figure 30a are assumed to be the spaces where the solvent phase was entrapped within the silica-rich phase. Another reason could be the formation of a O/W(hydrolyzed silanes)/O double emulsion. On the other hand, a late freezing, or premature phase separation (due, for instance, to a hydrolysate with high oligomerization degree and, therefore, larger viscosity and incompatibility with the polar solvent), results in an extensive coarsening of the structure (large characteristic size), as can be observed in Figure 30c, since there is freezing of the late stage of phase separation. In some cases (for off-critical compositions), it results even in the formation of particulate morphologies, i.e., breakage of the continuous gel network, inside the emulsion droplets. Figure 30b represents the case where freezing and phase separation by spinodal decomposition occur simultaneously. A high magnification image clearly shows its interconnected pore morphology (Figure 31). Figure 30d is an example of macroscopic two phase structure (isolated macropores), which is formed when liquid-liquid phase separation occurs and is followed by the gelation of the precipitated silica-rich phase, i.e., when the onset of gelation is very delayed compared to the onset of phase separation. It could also occur when gelation is ahead of the phase separation process, so that it is still in an early phase. Additionally, in these conditions, a large volume fraction of the gel-rich phase tends to result in isolated macropores, while a large volume fraction of the solvent-rich phase tends to result in a fragmented skeleton with dispersed particles.

Table 5 provides a list of the works (synthesis strategies) found in the literature for the achievement of sol-gel derived microspheres displaying macroporosity, due to phase separation. The most relevant applications for each case are also described.

**Figure 32:**
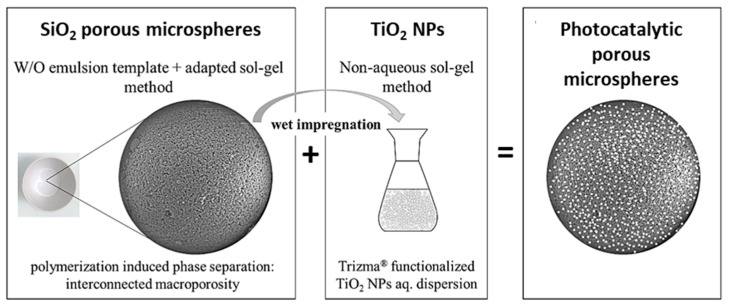
Methodology for preparing the photocatalytic porous microspheres: SiO_2_ microspheres loaded with TiO_2_ (anatase) NPs. Reprinted from the open access paper by A.C. Marques, M. Vale, D. Vicente, M. Schreck, E. Tervoort, M. Niederberger, “Porous Silica Microspheres with Immobilized Titania Nanoparticles for In-Flow Solar-Driven Purification of Wastewater”, Glob. Challenges 5 (2021) [3]. http://creativecommons.org/licenses/by/4.0/ (accessed on 19 March 2021).

The mechanical robustness, chemical inertness and transparency in the UV, associated with their macro and mesoporosity, make these microspheres an emerging platform for generating new and efficient engineered photocatalytic systems, by acting as a support and synergic structure where photocatalytic NPs are immobilized [3]. The inorganic SiO_2_ microspheres loaded with TiO_2_ NPs were prepared by direct wet impregnation with an aqueous dispersion of TiO_2_ NPs, as shown in Figure 32.

**Figure 33:**
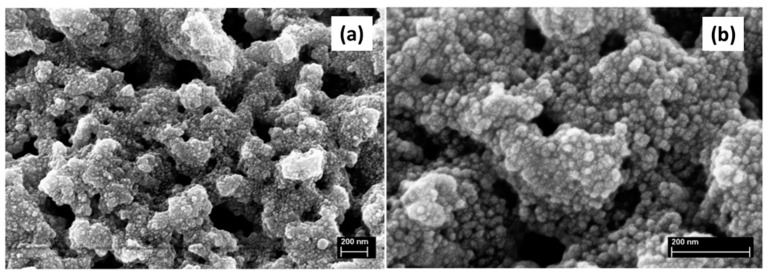
(**a**) SEM images of the MICROSCAFS loaded with TiO_2_ NPs; (**b**) magnified view of (**a**). Scale bar: 200 nm. Reprinted from the open access paper by A.C. Marques, M. Vale, D. Vicente, M. Schreck, E. Tervoort, M. Niederberger, “Porous Silica Microspheres with Immobilized Titania Nanoparticles for In-Flow Solar-Driven Purification of Wastewater”, Glob. Challenges 5 (2021) [3]. http://creativecommons.org/licenses/by/4.0/ (accessed on 19 March 2021).

This process resulted in the immobilization of TiO_2_ NPs on the microspheres, promoted by solvent evaporation and electrostatic interactions, forming a well attached, thin layer of NPs along the pores´ surface, as shown in Figure 33, not affecting the characteristic macroporosity of the spheres.

The resulting photocatalytic porous microspheres were inserted in a chamber of the continuous flow reactor set up and exposed to a solar simulator (1 Sun illumination). They were shown to allow a similar, or even better photocatalytic activity than other works in the literature (*k* of the order of 10^−2^ min^−1^ g^−1^), for methyl orange degradation, however with the advantage of the photocatalyst being easily removed from the reaction medium, since the NPs are grafted to a micron-size support material (MICROSCAFS), recyclable, and more appealing to a real, industrial application [3].

As future prospects, the composition of these microspheres (MICROSCAFS) can be tailored to enable synergistic phenomena together with the photocatalytic NPs. Their hierarchical porosity favors the light-harvesting and shortens the mass transfer/diffusion tracks, i.e., increases the capacity for mass transport and promotes the contact between the photocatalyst and pollutants. Moreover, they can be assembled into an infinity of shapes (e.g., in tubular, or serpentine reactors, columns, plates, etc.), which brings flexibility to the process, widens the possibility for new photocatalytic set-ups, and application in real-life conditions.

Beyond the works above referred on porous microspheres, it should be stressed that other shapes of products can also be achieved following the sol-gel/phase separation methodology. A similar formulation to that of the O phase of reference [125] was used to achieve hierarchically macro/mesoporous TiO_2_–SiO_2_ in the form of fibers [141]. These were fabricated by a typical electrospinning procedure, starting from a solution composed of PVP, DMF, acetic acid and Ti and Si precursors, TBOT and TEOS, respectively. Since no emulsion had to be prepared in this case, no surfactant, nor emulsification process was employed. The strong interaction between the TiO_2_-SiO_2_ oligomer and the carbonyl groups in PVP lead to the repulsion between oligomers and solvent (phase separation). DMF was the selected solvent in this case to assure that evaporation was slow enough, to delay the fibers´ solidification and to allow the coarsening of the phase-separated domains.

## 7. Conclusions

The pioneering works by Nakanishi and Soga (in the early nineties), on the enlightenment of phase separation induced by polymerization as a viable mechanism for porosity control in sol-gel systems, were at the origin of a variety of enabling materials for emerging technologies and advanced synthetic approaches towards tailored porous materials. This topic, which is extremely important for the development of advanced porous support materials, is still rapidly evolving, both in the field of monolithic materials and microspheres. The characteristic advantages of these materials are their interconnected porosity on the micrometer length scale, which takes place when the phase separation process, by spinodal decomposition, is occurring concomitantly to the gelation process. Structures with different coarsening extension (characteristic size) can be achieved depending on the timing between phase separation and sol-gel transition, as well as the stability of the heterogeneous phases and the volume fraction of the gel-rich and solvent-rich phases. Therefore, the control of all parameters resulting in faster sol-gel reactions, or that induce phase separation, is critical. Over the last thirty years, various synthesis strategies have been developed, resourcing different kinds of alkoxides and salt precursors, to reach hierarchical macroporous products made of a variety of materials, including silica (inorganic or hybrid, either of single oxide or multi-oxide composition), non-siliceous oxides (including C/oxides), and non-oxides (such as C), or MOFs. This review attempts to compile and critically discuss the most representative synthesis methodologies for macroporous microspheres and monolithic materials, as well as to give a major insight on microspheres with hierarchical macro/mesoporosity and their applications. The latter (microspheres) have not been represented in the literature so much as monoliths, but display immense potential for emergent applications. One example is their application in a new generation of photocatalytic systems, paving the way for environmental remediation in the form of a greener and more efficient water purification solution.

## Figures and Tables

**Table 1:** Overview of some of the latest developments on SiO_2_ macro and mesoporous hierarchically structured monoliths (inorganic and hybrid composition) prepared via sol-gel/phase separation processing.

Synthesis Strategy	Authors, Year Reference
Acidic sol-gel synthesis using a nonionic triblock copolymer (P123) as a phase separation inducer. Precursor: TMOS	Nakanishi et al., 1998 [28]
Acidic sol-gel synthesis without polymeric phase separation inducer additive, with methanol and water as common solvents. Precursor: bis(trimethoxysilyl)hexane (BTMH)	Nakanishi et al., 2002 [37]
Formamide-mediated sol-gel reaction. Acidic sol-gel synthesis without polymeric phase separation inducer additive, with methanol and water as common solvents, and formamide as additive polar solvent to promote gelation and phase separation. Precursor: TMOS and VTMS	Itagaki et al., 2003 [38]
Double-templating synthesis route: PEO as a phase separation inducer in combination with an ionic (CTAB) or nonionic surfactant (P123) as structure-directing agents at the nanometer range. Precursor: TEOS	Smått et al., 2003 [36]
Ethylene glycol-modified silane (precursor) in the presence of P123, for long-range ordering of the mesopores.	Hüsing et al., 2003 [43]
Propane-1,2-diol and glycerol-modified silane (precursor) in the presence of P123, for long-range ordering of the mesopores.	Hüsing et al., 2005 [44]
Acid/base two-step processing method to obtain macroporous PMSQ monoliths. No use of phase separation inducers. Precursor: MTMS	Dong et al., 2006 [39]
Acidic sol-gel synthesis using triblock copolymers (P123 and F127), and TMB as pore expander. Precursor: TMOS or bis(trimethoxysilyl)ethane (BTME)	Nakanishi et al., 2008 [34]
Weak acidic sol-gel synthesis using P123, and inorganic salts (NaCl, NaNO_3_ or Na_2_SO_4_) for ordered mesostructured and greater silica cross-linking. Precursor: TMOS	Zhong et al., 2009 [45]
Acid/base two-step processing method, using triblock copolymers (F127), to obtain macro/mesoporous PMSQ monoliths. Precursor: MTMS	Kanamori et al., 2011 [40]
Varied nonionic PEO-b-PPO-b-PEO triblock copolymers with different Mw and PO/EO ratios tested as phase separation inducers (PMSQ monoliths). Precursor: MTMS	Kurahashi et al., 2012 [42]

**Table 2:** Varied synthesis strategies for the achievement of SiO_2_-based multicomponent oxide monoliths, prepared via sol-gel/phase separation processing.

Composition	Synthesis Strategy	Authors, Year Reference
SiO_2_-TiO_2_	Acidic sol-gel synthesis using a phase separation inducer (HPAA). Precursors: TMOS and TBOT	Nakanishi et al., 1992 [47]
SiO_2_-TiO_2_	Acidic sol-gel synthesis using a phase separation inducer (PEG, 20,000 Mw) and two-step hydrolysis, or acac-complexation route. Precursors: TEOS and Ti salts and alkoxides	Ruzimuradov et al., 2012 [48]
SiO_2_-TiO_2_	Double-templating synthesis route: a phase separation inducer (PEG, 10,000 Mw), nonionic surfactant (P123), together with NH_4_F for further Ti incorporation in the framework. Precursors: TMOS and TiPOT	Yang et al., 2013 [49]
SiO_2_-ZrO_2_	Acidic sol-gel synthesis adding a phase separation inducer (PEO, 100,000 Mw) and specific alcohols to increase the domain size. Precursors: TMOS and zirconium tetra-2-propoxide (TPZR)	Takahashi et al., 1997 [50]
SiO_2_-Al_2_O_3_	Acidic sol-gel synthesis adding a phase separation inducer (PEO, 100,000 Mw). Precursors: TEOS and aluminum nitrate	Takahashi et al., 2001 [51]
SiO_2_-Al_2_O_3_	Acidic sol-gel synthesis adding a phase separation inducer (PEO, 10,000 Mw). Precursors: TMOS and aluminium sec-butoxide	Morai et al., 2004 [52]
SiO_2_-Al_2_O_3_	Double-templating synthesis route: a phase separation inducer (PEO, 100,000 Mw) and C_16_EO_10_ as the structure-directing agent. Precursors: TMOS and aluminum nitrate, or aluminum isopropoxide	Wu et al., 2007 [53]
SiO_2_-Al_2_O_3_	Double-templating synthesis route: aphase separation inducer (PEO, 10,000 Mw) and P123 as the structure-directing agent. Precursors: TMOS and aluminum nitrate	Yang et al., 2010 [54]
SiO_2_-Al_2_O_3_ (mullite)	Epoxide (propylene oxide, PO)-mediated sol-gel reaction + a phase separation inducer (PEO, 100,000 Mw) Precursors: TMOS and aluminum chloride	Guo et al., 2013 [55]
Ni/SiO_2_	Acidic sol-gel synthesis adding a phase separation inducer (PEO, 100,000 Mw). Precursors: TEOS and nickel nitrate	Nakamura et al., 2000 [56]
CuO/SiO_2_, NiO/SiO_2_	Acidic sol-gel synthesis adding a phase separation inducer (PEO, 10,000 Mw). Precursors: TMOS and nickel/copper nitrate	Zheng et al., 2006 [57]
SiO_2_-CaO	Acidic sol-gel synthesis adding a phase separation inducer (PEO, 100,000 Mw). Precursors: TMOS and calcium nitrate tetrahydrate (Ca(NO_3_)_2_·4H_2_O)	Marques et al., 2009 [58]
SiO_2_-CaO-P_2_O_5_	Acidic sol-gel synthesis, using two strategies: (i) PEO (100,000 Mw) as a phase separation inducer and urea; (ii) P123 as a phase separation inducer and 1,3,5-trimethylbenzene (TMB) as a pore expander (micelle-swelling agent). Precursors: TMOS, calcium nitrate tetrahydrate (Ca (NO_3_)_2_·4H_2_O), and triethyl orthophosphate	Marques et al., 2007; [59]
MgO–Al_2_O_3_–SiO_2_ (cordierite)	Epoxide (PO)-mediated sol-gel reaction + a phase separation inducer (PAAm). Precursors: TMOS, magnesium chloride and aluminum chloride	Guo et al., 2014 [60]
Al_2_O_3_ -SiO_2_-TiO_2_	Formamide-mediated sol-gel reaction + a phase separation inducer (PEO). Precursors: TEOS, TiPOT, aluminum nitrate nonahydrate	Sun et al., 2016 [61]

**Table 3:** Specific examples of synthesis strategies for the achievement of non-siliceous single oxide porous monoliths, prepared via sol-gel/phase separation processing.

Composition	Synthesis Strategy	Authors, Year Reference
TiO_2_	Formamide-mediated sol-gel reaction (acidic medium) + a phase separation inducer (PEO, 300,000 and 100,000 Mw). Precursor: colloidal anatase-type TiO_2_ (7 nm size particles, aqueous dispersion, pH 1.7)	Fujita et al., 2004 and 2006 [66,67]
	Formamide-mediated sol-gel reaction (strongly acidic medium, HCl) + low temperatures. Precursor: TiPOT	Konishi et al., 2006 [68]
	Nearly neutral sol-gel synthesis using a chelating agent (EtAcAc) + mineral salt (NH_4_NO_3_) + a phase separation inducer (PEO, 10,000 Mw). Precursor: TiPOT	Hasegawa et al., 2010 [69]
	NMF-mediated sol-gel reaction (strongly acidic medium, HCl) + low temperatures + a phase separation inducer (PEO, 10,000 Mw). Precursor: TiPOT	Konishi et al., 2009 [71]
	Reaction rate controlling additives: strongly acidic sol-gel synthesis (HCl) + a chelating agent (acetic acid). Precursor: TiPOT	Backlund et al., 2007 [73]
	Formamide-mediated sol-gel reaction (acidic medium) + a phase separation inducer (PVP, 10,000 Mw) + EG as a chelating agent. Precursor: TiOSO_4_	Li et al., 2013 [72]
ZrO_2_	NMF-mediated sol-gel reaction (strongly acidic medium, HNO_3_) + low temperatures + a phase separation inducer (PEO, 35,000 Mw). Precursor: Zirconium isopropoxide	Konishi et al., 2008 [75]
	Epoxide (PO)-mediated sol-gel reaction + a phase separation inducer (PEO, 1,000,000 Mw) Precursors: Zirconium oxychloride octahydrate (ZrOCl_2_·8H_2_O)	Guo et al., 2015 [78]
Magnesia and yttria stabilized ZrO_2_	Epoxide (PO)- and NMF-mediated sol-gel reaction + a phase separation inducer (PEO, 1,000,000 Mw) Precursors: anydrous zirconium chloride (ZrCl_4_) and MgCl_2_·6H_2_O or YCl_3_·6H_2_O	Wu et al., 2014 [77]
Yttria stabilized ZrO_2_ (YZA)	Epoxide (PO)- and formamide-mediated sol-gel reaction + a phase separation inducer (PEO, 300,000 Mw) + EG as a chelating agent Precursors: Zirconium oxychloride (ZrOCl_2_·8H_2_O) and yttrium chloride (YCl_3_·6H_2_O)	Guo et al., 2016 [79]
Al_2_O_3_	Epoxide (PO)-mediated sol-gel reaction + a phase separation inducer (PEO, 1,000,000 Mw) Precursor: AlCl_3_·6H_2_O	Tokodume et al., 2007 [76]
Cr_2_O_3_	Epoxide (PO)-mediated sol-gel reaction + a phase separation inducer (HPAA) + urea. H.T under air atmosphere. CrN-C and Cr_3_C_2_-C composites will form if H.T. is under N_2_ atmosphere. Precursor: chromium chloride hexahydrate CrCl_3_·6H_2_O	Kido et al., 2014 [81]
MgO	Epoxide (PO)-mediated sol-gel reaction + a phase separation inducer (PVP, 100,000 Mw). Precursor: Magnesium chloride hexahydrate (MgCl_2_·6H_2_O)	Li et al., 2016 [93]
	Epoxide (PO)-mediated sol-gel reaction + a phase separation inducer (PVP, 10,000 Mw) + 1,3,5-benzenetricarboxylic acid to preserve the fine crystallite size. Precursor: Magnesium chloride hexahydrate (MgCl_2_·6H_2_O)	Lu et al., 2019 [83]
ZnO	Epoxide (PO)-mediated sol-gel reaction + citric acid to coordinate to Zn cations and promote phase separation. 1,2-epoxybutane was also tested and found to have lower solubility and compatibility than PO, enhancing phase separation. Precursor: Zn(NO_3_)_2_·6H_2_O	Lu et al., 2019 [90]
CoO_2_, CuO_2_, MnO_2_	Epoxide (epichlorohydrin)-mediated sol-gel reaction (acidic medium) + a phase separation inducer (PEO 100,000–600,000 Mw and/or PVP 40,000 Mw). Precursors: metal bromides (MBr_2_, with M = Cu,Co,Mn), which transform into brominated metal alkoxides, by reaction with epichlorohydrin	Lu et al., 2020 [92]
Fe_2_O_3_	Epoxide (PO and trimethylene oxide)-mediated sol-gel reaction + a phase separation inducer (PAAm, 10,000 Mw). Precursor: Iron(III) chloride hexahydrate (FeCl_3_·6H_2_O)	Kido et al., 2012 [82]
Fe_3_O_4_	Epoxide (PO)-mediated sol-gel reaction + a phase separation inducer (PAAm, 10,000 Mw). Precursor: Iron(II) chloride tetrahydrate (FeCl_2_·4H_2_O)	Wang et al., 2020 [94]

**Table 4:** Specific examples of synthesis strategies for the achievement of non-siliceous multi-oxide and non-oxide porous monoliths, prepared via sol-gel/phase separation processing.

Composition	Synthesis Strategy	Authors, Year Reference
Aluminum phosphate AlPO_4_	Epoxide (PO)-mediated sol-gel reaction + a phase separation inducer (PEO, 10,000 Mw). Precursor: AlCl_3_·6H_2_O and H_3_PO_4_	Li et al., 2013 [97]
Metal zirconium phosphates (MZr_2_(PO_4_)_3_)	Acidic sol-gel synthesis adding a phase separation inducer (PEO, 35,000 Mw and PAAm, 10,000 Mw). Precursors: ZrOCl_2_·8H_2_O, H_3_PO_4_, and several metal chlorides	Zu et al., 2016 [98]
Lithium zirconate Li_2_ZrO_3_	Epoxide (PO)-mediated sol-gel reaction + a phase separation inducer (PEO, 1,000,000 Mw). Precursors: ZrOCl_2_·8H_2_O and lithium acetate dehydrate (LiOAc·2H_2_O)	Guo et al., 2017, [84]
Barium zirconate BaZrO_3_	Epoxide (PO)- and formamide-mediated sol-gel reaction + a phase separation inducer (PEO, 300,000 Mw) + EG as a chelating agent Precursors: Zirconium oxychloride (ZrOCl_2_·8H_2_O) and barium chloride dihydrate (BaCl_2_·2H_2_O)	Guo et al., 2017 [80]
Zirconium titanate ZrTiO_4_	*N*-methyl formamide-mediated sol-gel reaction (strongly acidic medium, HNO_3_, ice cooling) + low temperatures + a phase separation inducer (PEO, 100,000 Mw). Precursors: ZrOCl_2_·8H_2_O and TiPOT	Sun et al., 2019 [89]
CaTiO_3_, SrTiO_3_ and BaTiO_3_ perovskite	Impregnation of preformed macroporous TiO_2_ monoliths, with alkaline-earth metal ions, in urea solution. Nearly neutral sol-gel synthesis using a chelating agent (EtAcAc) + mineral salt (NH_4_Cl) + a phase separation inducer (PEO, 10,000 Mw). Precursors: TiPOT, CaCl_2_·2H_2_O, SrCl_2_·6H_2_O, and BaCl_2_·2H_2_O	Ruzimuradov et al., 2011 [99]
**La_2_Zr_2_O_7_**	Epoxide (PO)- and formamide-mediated sol-gel reaction + a phase separation inducer (PEO, 300,000 Mw). Precursors: Zr(NO_3_)_4_·5H_2_O, ZrOCl_2_·8H_2_O, La(NO_3_)_3_·6H_2_O, and LaCl_3_·6H_2_O	Wang et al., 2016 [87]
Aluminum titanate Al_2_TiO_5_	Formamide-mediated sol-gel reaction + a phase separation inducer (PEO, 100,000 Mw) + a chelating agent (citric acid) and ice cooling. Precursors: AlCl_3_·6H_2_O and titanium tetrabutoxide (Ti(OBu)_4_)	Guo et al., 2015 [100]
ZnFe_2_O_4_ spinel	Epoxide (PO)-mediated sol-gel reaction + a phase separation inducer (PAAm, 10,000 Mw). Precursors: FeCl_3_·6H_2_O and ZnCl_2_ (Zn/Fe, R = 0.50 in molar ratio) (low-valence elements)	Kido et al., 2013 [95]
NiAl_2_O_4_ and CoAl_2_O_4_ spinel	Epoxide (PO)-mediated sol-gel reaction + a phase separation inducer (PEO, 900,000 Mw). Precursors: AlCl_3_·6H_2_O, NiCl_2_· 6H_2_O, or CoCl_2_·6H_2_O (low-valence elements).	Herwig et al., 2018 [85]
ZnAl_2_O_4_ spinel	Epoxide (PO)-mediated sol-gel reaction + a phase separation inducer (PEO, 1,000,000 Mw). Precursors: ZnCl_2_ and AlCl_3_·6H_2_O (low-valence elements)	Guo et al., 2017 [88]
CoMn_2_O_4_ spinel	Epoxide (epichlorohydrin)-mediated sol-gel reaction (acidic medium) + a phase separation inducer (PEO 400,000 Mw and/or PVP 10000, 40000, and 360,000 Mw). Precursors: metal bromides (MBr_2_, with M=Co, Mn), which transform into brominated metal alkoxides, by reaction with epichlorohydrin (low-valence elements)	Lu et al., 2020 [101]
Transition metal hydroxides, ZnOH, CuOH, MnOH and binary compositions	Epoxide (PO)-mediated sol-gel reaction + a phase separation inducer (HPAA, 100000Mw). Precursors: ZnCl_2_, CoCl_2_·6H_2_O, NiCl_2_·6H_2_O, MnCl_2_·4H_2_O, and FeCl_2_·4H_2_O	Liu et al., 2020 [96]
Carbon (C)/TiO_2_	Formamide-mediated sol-gel reaction + a phase separation inducer and C source (PVP, 10,000 Mw) + ethylene glycol, solvent, and a chelating agent. Precursor: TiOSO_4_·xH_2_O	Zhu et al., 2015 [102]
Resorcinol formaldehyde (RF) and C	Acidic sol-gel synthesis, using surfactant F127 as a phase separation inducer and pore directing agent, TMB and benzyl alcohol BzOH (cosurfactant) as micelles´ swelling agents, and TEG as a compatible solvent with RF/F127 oligomers to suppress excess phase separation. Precursors: resorcinol (and formaldehyde)	Hasegawa et al., 2016 [103]
RF and C	Acidic sol-gel synthesis, using surfactant F127, TMB, BzOH, TEG, and inorganic salt KCl. Precursors: resorcinol (and formaldehyde)	Hasegawa et al., 2020 [27]
RF/TiO_2_ and C/TiO_2_	Acidic sol-gel synthesis, using surfactant F127, TMB, BzOH, and TEG. Precursors: resorcinol (formaldehyde) and EGMT	Schoiber et al., 2021 [104]
Metal organic frameworks (MOFs)	Sol-gel synthesis and self-assembly-induced phase separation, using PPG (1000 Mn), solvent DMF, and acetic acid as a mediator for reorganization of the microstructure Precursors: ZrOCl_2_·8H_2_O and 2-aminoterephthalic acid (BDC-NH_2_) as an organic linker	Hara et al., 2019 [105]

**Table 5:** Compilation of specific examples of macroporous microspheres and their synthesis strategies based on the emulsion method combined with sol-gel transition and phase separation.

Characteristics of the Microspheres		Synthesis Strategy/Applications	Author, Year Reference
Incontinuous multicavities	TiO_2_, ZrO_2_ and Al_2_O_3_ hollow microspheres with incontinuous multicavities	(1) O/W emulsion (O phase: metal alkoxide, EtAcAc, 1-octanol, Span 80, PVP; W phase: DI water and surfactants SDS and OP-10) (2) nucleation growth phase separation (3) nearly neutral sol-gel synthesis using a chelating agent (EtAcAc) + a phase separation inducer (PVP 30,000 Mw) and Span 80 as a phase separation and spherical morphology stabilizer. Precursor: TBOT, or zirconium propoxide, or aluminum tri-sec-butoxide Applications: liquid chromatography and heat insulation	Cai et al., 2014 [125]
Macro/ mesoporosity	Inorganic SiO_2_ microspheres	(1) W/O emulsion (W phase: hydrolyzed silane solution using a molar ratio H_2_O/precursor ~ 11, F127 as surfactant and PEO 20000Mw as macropore former); O phase: paraffin oil and Span 80 (2) pH induced rapid colloid aggregation method, by addition of triethylamine (TEA): sol-gel transition in parallel with phase separation. (3) calcination Precursors: TEOS No specific application tested	Yang et al., 2006 [127]
	Inorganic SiO_2_ microspheres	(1) W/O emulsion (W phase: hydrolyzed silane solution using a molar ratio H_2_O/precursor ~18, and PEO 100,000 Mw); O phase: paraffin oil and Span 80 (2) growth of siloxane oligomers: sol-gel transition in parallel with phase separation (3) burning of PEO leads to mesopores and fluid phase evaporation leads to macropores. (4) calcination Precursors: TEOS Applications: SiO_2_ porous microspheres covalently bonded with octadecyl tested for liquid chromatography (fast separation)	Shi et al., 2008 [112]
	Waxberry-like and ethyl-bridged hybrid SiO_2_ microspheres	(1) W/O emulsion (W phase: hydrolysed silanes solution using a molar ratio H_2_O/precursors ~ 25 and PEO 10,000 Mw); O phase: petroleum ether, Triton X-100 and Span 80 (2) growth of siloxane oligomers: sol-gel transition in parallel with phase separation (3) burning of PEO leads to mesopores and fluid phase evaporation leads to macropores Precursors: TEOS and BTME Applications: Alkali resistant carrier with fast mass transfer property; tested for catalysis and liquid chromatography	Li et al., 2019 [110]
	Hybrid, epoxy functionalized and inorganic SiO_2_ microspheres	(1) W/O emulsion (W phase: water; O phase: decahydronaphthalene and Span 80) (2) addition of the hydrolysed silanes solution (with molar ratio H_2_O/precursors = 4.7) to the W phase (3) growth of silica-epoxy oligomers; sol-gel transition in parallel with phase separation inside the emulsion droplets; no need for templates or a phase separation inducer additives, no need for calcination to obtain meso and macroporosity. Study of synthesis parameters [2]. Precursors: TEOS and GPTMS (1/0.89 molar ratio) Applications: (i) Biocide Econea^®^ immobilization within the porous microspheres (grafting) for antifouling applications [140]; (ii) TiO_2_ NPs immobilization within the porous microspheres for solar light driven photocatalysis, using a continuous flow reactor [3]	Loureiro et al., 2018 [140] Vale et al., 2020 [2] Marques et al., 2021 [3]

## Abbreviations

Acac	Acetylacetone
ATR	Attenuated total reflection
BTME	1,2-Bis(trimethoxysilyl)ethane
BTMH	bis(trimethoxysilyl)hexane
BzOH	Benzyl alcohol
CTAB	n-hexadecyltrimethylammonium bromide
CTAC	n-hexadecyltrimethylammonium chloride
Decalin	Decahydronaphthalene
DEG	Diethylene glycol
DI	Deionized
DMDMS	Dimethoxydimethylsilane
DMF	N,N-dimethylformamide
DTA	Differential thermal analysis
EDA	Ethylenediamine
EG	Ethylene glycol
EGDMA	Ethylene glycol dimethacrylate
EGMT	(Ethylene glycol)-modified titanate
EO	Ethylene oxide
EtAcAc	Ethyl acetoacetate
FTIR	Fourier Transform Infrared
GMA	Glycidyl methacrylate
GPTMS	(3-glycidyloxypropyl) trimethoxysilane
HDTMS	Hexadecyltrimethoxysilane
HLB	Hydrophilic-lipophilic balance
HPAA	Poly(acrylic acid)
LCST	Lower critical solution temperature
MOFs	Metal–organic frameworks
MTES	Methyltriethoxysilane
MTMS	Methyltrimethoxysilane
NaPSS	Poly(sodium-4-styrene sulfonate)
NMF	N-methylformamide
NMR	Nuclear magnetic resonance
nOTES	octyltriethoxysilane
NPs	nanoparticles
O	oil phase
O/W	oil-in-water
OP-10	alkylphenol ethoxylate surfactant
P123	triblock copolymer surfactant
PAAm	poly(acrylamide)
PEG	Polyethylene glycol
PEO	Poly(ethylene oxide)
PhTMS	Phenyltrimethoxysilane
PMSQ	Polymethylsilsesquioxane
PO	Propilene Oxide
PPG	Polypropylene glycol
PS	Polystyrene
PVP	Poly(vinylpyrrolidone)
RE	Rare earth
RF	Resorcinol–formaldehyde
RT	Room temperature
SAXS	Small-angle x-ray scattering
SDS	Sodium dodecyl sulfate
TBOT	Titanium tetrabutoxide
TEA	Triethylamine
TEG	Triethylene glycol
TEOS	Tetraethyl orthosilicate
t_g_	gelation time
TG	Thermogravimetry
TiPOT	Titanium isopropoxide
TMB	Trimethyl benzene
TMOS	Tetramethoxysilane
TOA	Trioctylamine
TPZR	zirconium tetra-2-propoxide
UCST	Upper critical solution temperature
VTMS	Vinyltrimethoxysilane
W	water phase
W/O	water-in-oil
XRD	X-ray diffraction
YAG	Yttrium aluminum garnet
YZA	yttria-stabilized zirconia
